# Disordered Rock-Salt Type Li_2_TiS_3_ as Novel Cathode for LIBs: A Computational Point of View

**DOI:** 10.3390/nano12111832

**Published:** 2022-05-27

**Authors:** Riccardo Rocca, Mauro Francesco Sgroi, Bruno Camino, Maddalena D’Amore, Anna Maria Ferrari

**Affiliations:** 1 Department of Chemistry and NIS, University of Turin, 10125 Torino, Italy; maddalena.damore@unito.it; 2 Centro Ricerche FIAT S.C.p.A., 10043 Orbassano, Italy; mauro.sgroi@crf.it; 3 Department of Chemistry, Imperial College, London SW7 2AZ, UK; b.camino13@imperial.ac.uk

**Keywords:** DFT, Li-ion batteries, crystal, solid solutions, fingerprint

## Abstract

The development of high-energy cathode materials for lithium-ion batteries with low content of critical raw materials, such as cobalt and nickel, plays a key role in the progress of lithium-ion batteries technology. In recent works, a novel and promising family of lithium-rich sulfides has received attention. Among the possible structures and arrangement, cubic disordered Li_2_TiS_3_ has shown interesting properties, also for the formulation of new cell for all-solid-state batteries. In this work, a computational approach based on DFT hybrid Hamiltonian, localized basis functions and the use of the periodic CRYSTAL code, has been set up. The main goal of the present study is to determine accurate structural, electronic, and spectroscopic properties for this class of materials. Li_2_TiS_3_ precursors as Li_2_S, TiS_2_, and TiS_3_ alongside other formulations and structures such as LiTiS_2_ and monoclinic Li_2_TiS_3_ have been selected as benchmark systems and used to build up a consistent and robust predictive scheme. Raman spectra, XRD patterns, electronic band structures, and density of states have been simulated and compared to available literature data. Disordered rock-salt type Li_2_TiS_3_ structures have been derived via a solid solution method as implemented into the CRYSTAL code. Representative structures were extensively characterized through the calculations of their electronic and vibrational properties. Furthermore, the correlation between structure and Raman fingerprint was established.

## 1. Introduction

Nowadays, lithium-ion batteries (LIBs) are widely employed in electrified vehicle and several other applications, and for this reason the market demand for LIBs is increasing very rapidly. High energy and power density are two features required from the materials used in LIBs. In this context, several lithium metal oxides have been studied and employed as cathode materials: lithium cobalt oxide (LCO), lithium manganese oxide (LMO), lithium nickel cobalt aluminum oxide (NCA), and lithium nickel manganese cobalt oxide (NMC). Most of them are characterized by a layered structure and operate by reversible intercalation and deintercalation of lithium ions during the discharge and charge processes, respectively. The main problem that arises from the use of these oxides is the high content of critical elements, nickel and especially cobalt. This issue has been widely addressed in recent years by proposing alternative chemical formulations.

Among them, a new family of lithium-rich sulfide materials was reported as novel and promising cobalt-nickel free cathodes. In particular, Li_2_TiS_3_ exhibits electrochemical performances comparable to the lithium-rich oxides, thanks to the large reversible capacity during cycling [1]. Similar systems are also reported with a variable selenium content as doping agent, showing comparable features with in respect the Li_2_TiS_3_ system [2,3]. In our work, we will focus only on Li_2_TiS_3_ without doping agents. The interest this kind of material has received is very high. This is due mainly to their low cost, as titanium is a more abundant and a less critical metal then cobalt and nickel. The most stable structure for Li_2_TiS_3_ is the layered monoclinic one, widely reported in the literature. However, the disordered cubic rock-salt type structure has recently attracted more and more attention because of its great capacities higher than 400 mAh∙g^−1^ upon cycling between 1.5–3 V vs. Li^+^/Li [4,5], which is larger than the capacity shown by layered monoclinic structure [1].

The synthesis of cubic Li_2_TiS_3_ is not a straightforward task since different products can be obtained, such as LiTiS_2_ and monoclinic Li_2_TiS_3_. Furthermore, the rock-salt structure of Li_2_TiS_3_ has been scarcely studied and characterized, and only a few studies are available in the literature [6,7]. Accurate calculations based on density functional theory (DFT) represent an unrivaled tool for the prediction of structural, electronic, and vibrational properties necessary for the identification and the characterization of novel compounds as it is the case of the cubic Li_2_TiS_3_. In particular, calculations of XRD patterns are of primary relevance since they can directly characterize the crystal structure of the material. Computed Raman spectroscopy represents another extremely powerful instrument, widely employed for materials characterization. This is because it can provide specific fingerprints associated to a certain atomic arrangement in the material, thus it is useful to discriminate between different structures and different chemical environments within similar structures [8,9,10,11,12]. Finally, an accurate description of the electronic structure of a material is a mandatory goal, necessary for a deep understanding of the system at the atomic level and of the intrinsic relationship between its structure and properties.

The need arises, therefore, to develop a robust and accurate computational scheme, reliable in predicting the properties of a cubic Li_2_TiS_3_. To achieve this, well-known and well characterized materials, Li_2_S, TiS_2_, TiS_3_, LiTiS_2_, and monoclinic Li_2_TiS_3_ (precursors or byproducts in the cubic Li_2_TiS_3_ synthesis) have been used for benchmark calculations to predict structural properties, Raman spectra, XRD patterns, and electronic structures (band structure and density of states). They have been simulated and compared with the data reported in the literature.

The cubic Li_2_TiS_3_ can be formally derived from a Li_2_S-TiS_2_ solid solution where 33% of Li is replaced by Ti in the 4a Wickoff lattice positions, to yield the stoichiometry of Li_2_TiS_3_. The number of configurations derived by the substitution of Ti with Li has been computed with a solid solution tool implemented in the CRYSTAL code. The analysis of all these configurations has highlighted the presence of two macro-groups of structures characterized by unique structural, electronic, and spectroscopic properties. Selected structures have indeed accurately characterized, and a reasonable model for Li_2_TiS_3_ has been finally put forward. Properties of cubic Li_2_TiS_3_ have been compared with those of its well-known monoclinic structure.

## 2. Computational Details

All calculations were performed by employing the periodic CRYSTAL17 code [13], based on DFT Hamiltonians and localized Gaussian type basis functions. The B3LYP [14,15] “hybrid” functional was chosen for most of the calculations in combinations with, split valence triple-zeta basis sets plus polarization (TZVP). For layered systems characterized by a weak interaction between layers semiempirical DFT-D2 * approach based on Grimme’s empirical corrections modified for solid systems [16] combined with the B3LYP functional (hereafter referred to as B3LYP-D2 *) was adopted. Two different basis sets were selected, basis *set A* and basis *set B*. Basis *set A* consists of Peintinger, Pob_TZVP_rev2 [17], for Li, 6211-2 [4s, 1p], and S, 73211-5111-1 [5s,4p,1d], and for Ti a small core Hay and Wadt pseudopotental [18] combined with a 411-311 [3sp,3d] for valence electrons. Basis *set B* consists of Ahlrichs TZVP [19] functions, characterized by: S with 73211-6111-1 [5s,4p,1d] Ti with 842111-631-411 [6s,3p,3d], and Li with 6211-2 [4s,1p] [20]. In the CRYSTAL code, the truncation criteria of the Coulomb and exchange infinite lattice series are controlled by five thresholds, which were set to 7 (T1–T4) and 14 (T5). The SCF convergence threshold for the energy was set to 10^−8^ Hartree for the structural optimization, whereas for vibrational frequency calculations, it was set to 10^−10^. The reciprocal space sampling is based on a regular Pack–Monkhorst sub-lattice grid centered at the Γ point, with shrinking factor 6 and 12 along each of the reciprocal lattice vectors, which corresponds to a number of **k**-points in the irreducible part of the first Brillouin zone that varies from 6 for more symmetric spatial group to 12 for systems with low symmetry.

Raman spectra were computed with the harmonic approximation, and XRD patterns were computed with well-assessed tools available in the CRYSTAL code [13]. For electron band calculations, the sampling of the first Brillouin zone of the reciprocal space was performed with reference to the Bilbao Crystallographic Server [21,22] and to the ones reported in relevant literature [23]. The **k**-points grid was built as described above.

Models for cubic Li_2_TiS_3_ have been obtained by employing the solid solution approach as implemented in the CRYSTAL code through the CONFRAND keyword. The starting structure was obtained from experimental data provided by Celasun et al. [3,24], reported in Table S1 in the Supplementary Materials. A 3 × 3 × 3 supercell of the primitive cell was used. This cell represents the smallest cell with the exact stoichiometry for Li_2_TiS_3_. In this system, 54 atoms are present, Ti and Li atoms can occupy 27 possible equivalent sites, 18 of them are occupied by Li atoms, and 9 by Ti atoms. The number of possible configurations was generated by replacing 9 Ti out of the 27 Li in the starting structure. The binomial coefficient $\left(\begin{array}{c} 27\\ 9 \end{array}\right)=4,686,825$ gives us all the possible configurations, which reduces to 4023 when symmetry is taken into account [25]. Symmetry equivalent structures can be obtained from each other by applying symmetry operators, and they indeed share the same energy and properties.

The 4023 symmetry unique structures were rationalized according to a geometrical criterion involving Ti-Ti average distances and related standard deviations. Details on the statistical analysis performed on all structures and its results can be easily found online according to Ref. [26]. This analysis was made possible thanks to the crystal_functions, a python interface to the CRYSTAL code available at [27]. In the present paper, we attempted to identify a possible correlation between stability, electron properties, and local arrangement of Ti atoms. The accurate computational methodology (B3LYP/TZVP) described at the beginning of the section, was applied to selected structures. These structures were fully characterized by computing their structural and electron properties, XRD patterns, and Raman spectra.

## 3. Results

### 3.1. Sulfide Compounds Properties

The performance of the selected methodology (B3LYP in combination with basis sets *A* and *B*) was assessed on Li_2_S, TiS_2_, TiS_3_, LiTiS_2_, and monoclinic Li_2_TiS_3_ structures. The relaxed geometry, electron properties, XRD pattern, and Raman spectra were computed and compared with available experimental data. Structural data was obtained from the Inorganic Crystallographic Structure Dataset (ICSD) [28], except for crystallographic data of monoclinic Li_2_TiS_3_ that was taken from a previous study by Flamary et al. [1]. Relevant structural parameters for the investigated compounds are reported in Table 1.

In Table 1, a general reasonable agreement between computed and experimental structures can be observed. Discrepancies variate from 1 to 2% from the experimental data. However, for layered structures as TiS_2_ and TiS_3_, the weak interaction between layers is not adequately taken into account by the B3LYP functional, because common hybrid GGA functionals cannot account for long-range correlation and hence are unable to well describe dispersive interactions. The DFTD2 methods may partly overcome these limitations, these forces were considered by using the B3LYP-D2 * functional.

Computed band gaps are also in reasonable agreement with the experimental counterpart despite a slight overestimation, a well-known issue for this functional [29,30], see Table 1. Band structures and the density of states, shown in Figure S1 of Supplementary Materials, are in line with previous reports; the top of the valence band is characterized by sulfur 3 sp states, whereas the bottom of conduction band is dominated by electron states from Li and/or Ti atoms.

Simulated XRD patterns satisfactorily match the experimental data (retrieved from the ICSD database), except for small deviations that become more significant at higher angles, see Figure S2 of Supplementary Materials. For instance, in the case of Li_2_S at high angles, the simulated pattern is lower than the experimental one only by 0.5°, while at higher angles the difference increases and the experimental peak at 89° can be found in the calculated one at 87°.

**Table 1:** Structural parameters and band gap for Li_2_S, TiS_2_, TiS_3_, LiTiS_2_, and monoclinic Li_2_TiS_3_. Experimental structural data are retrieved from ICSD, band gap from the reference indicated in the table.

	Set A	Set B	Experimental
**Li_2_S—Fm—3m**			
**Cell Parameter a (A)**	5.81	5.81	5.72
**Li-S distance (A)**	2.51	2.51	2.47
**Band Gap (eV)**	5.32	5.41	3.8–4.0 [31]
**TiS_2_—P—3m1**			
**Cell Parameter a,b (A)**	3.46	3.46	3.41
**Cell Parameter c (A)**	5.89	6.00	5.70
**Band Gap (eV)**	1.51	0.98	0.5–1.0 [32,33]
**TiS_3_—P 21/m**			
**Cell Parameter a (A)**	5.19	5.19	4.95
**Cell Parameter b (A)**	3.46	3.45	3.40
**Cell Parameter c (A)**	12.80	12.93	8.77
**β (°)**	94.81	96.33	97.32
**Band Gap (eV)**	1.85	1.47	1.0–1.2 [34,35]
**LiTiS_2_—P—3m1**			
**Cell Parameter a, b (A)**	3.48	3.49	3.45
**Cell Parameter c (A)**	6.23	6.32	6.18 [36]
**Band Gap (eV)**	conductor	conductor	conductor
**Li_2_TiS_3_—C2/m**			
**Cell Parameter a (A)**	6.28	6.26	6.15
**Cell Parameter b (A)**	10.88	10.84	10.67
**Cell Parameter c (A)**	6.45	6.43	6.32
**β (°)**	109.2	109.2	109.0 [1]
**Band Gap (eV)**	2.40	2.06	-

**Figure 1:**
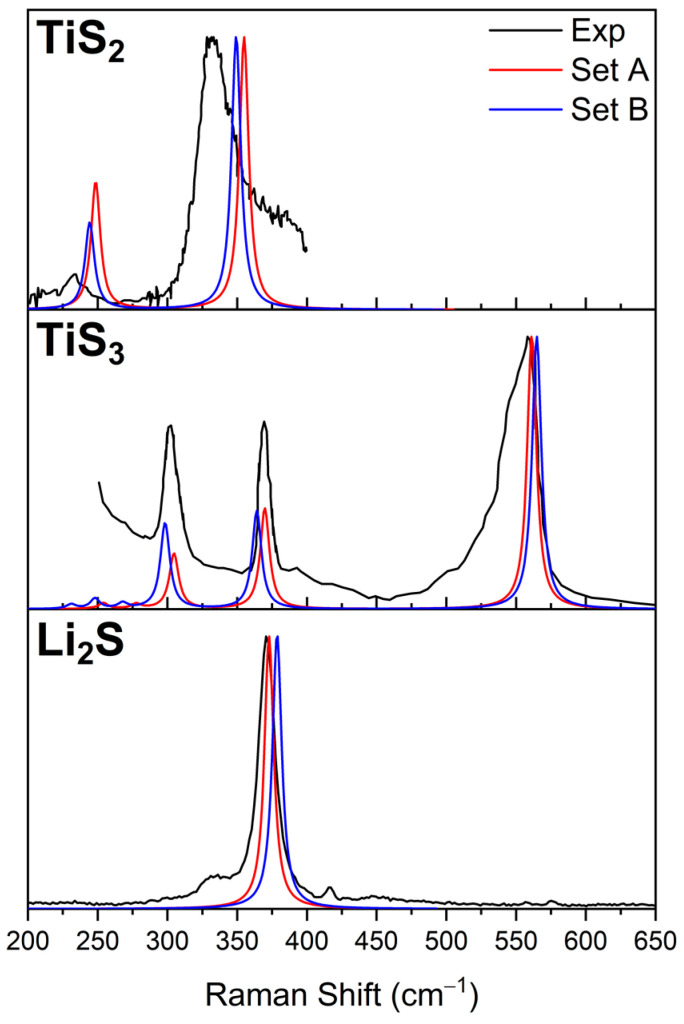
Raman spectra of Li_2_S, TiS_2_, and TiS_3_: experimental (black), calculated with basis *set A* (red), and calculated with basis *set B* (blue). Li_2_S has been simulated at B3LYP, whereas B3LYP-D2 * level of computation has been adopted for TiS_2_ and TiS_3_.

Simulated and experimental Raman spectra for Li_2_S, TiS_2_, and TiS_3_ are reported in Table 2 and Figure 1. Li_2_S is characterized by one peak at 375 cm^−1^ (B3LYP/Basis B) corresponding to the Li-Ti stretching mode; TiS_2_ is characterized by two peaks at 244 and 349 cm^−1^ (B3LYP-D2 */Basis B) corresponding to the E_g_ and A_1g_ in plane and out of plane Ti-S deformation in the TiS_2_ layers; TiS_3_ is characterized by three peaks at 298, 364, and 565 cm^−1^ (B3LYP-D2 */Basis B) assigned to the internal vibrations of polyhedral TiS_6_ units and the S-S stretching modes, respectively [37]. The agreement between computed and experimental Raman spectra is excellent; basis *sets A* and *B* perform in similar ways even if *set B* proved to better reproduced the experimental data with the average of the absolute deviation from experimental Raman peaks equal to 5.5 cm^−1^.

**Figure 2:**
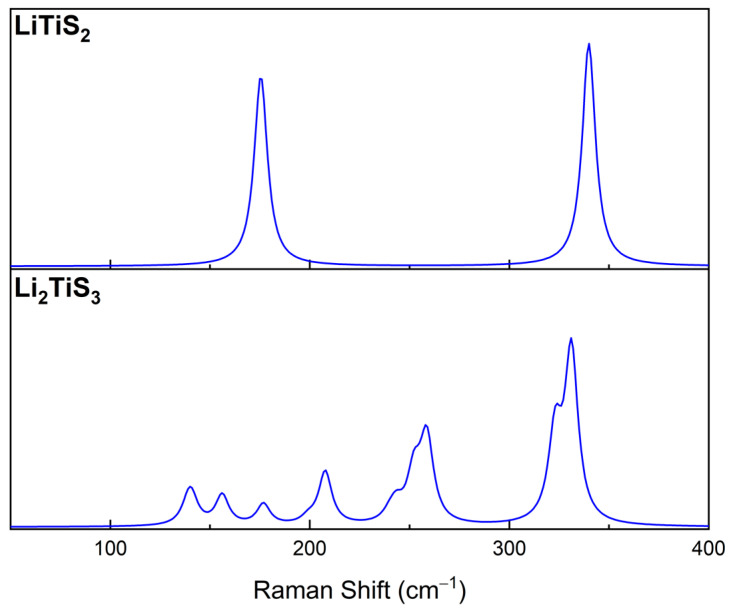
Raman spectra for LiTiS_2_ and Li_2_TiS_3_ computed with the B3LYP functional and basis *set B*.

Raman spectra for LiTiS_2_ and monoclinic Li_2_TiS_3_ are also reported in Figure 2. However, for these systems, experimental counterparts were not found in literature. LiTiS_2_ is characterized by E_g,_ and A_1g_ peaks at 340 and 125 cm^−1^ that correspond to in plane and out of plane Ti-S deformation in the TiS_2_ layers; similar motion involving Li atoms falls at 189 and 319 cm^−1^, but they are not Raman active. Regarding the monoclinic form of Li_2_TiS_3_, Raman spectra are characterized by four main regions. The vibrational modes mainly associated to lithium atoms in the direction perpendicular to the lithium layers, can be found at high wavenumbers in the range 320–330 cm^−1^, whereas the peaks around 250 cm^−1^ are related to the vibrations of lithium atoms perpendicular to the same layer. Inside the monoclinic structure, it is possible to recognize 4-member and 12-member rings composed by titanium and sulfur atoms, the stretching vibrations of these “rings” are associated to the peak at 210 cm^−1^. The ring structures also influence the lowest wavenumber Raman peaks; in fact, the two peaks at 147 and 152 cm^−1^ are related to rings deformation. The previous analysis of Raman spectra shows that this technique plays a key role in the characterization of these materials. This technique was, indeed, able to reproduce the specific fingerprints of each specific structure.

### 3.2. Disordered Cubic Li_2_TiS_3_

Aiming to simulate the stoichiometric Li_2_TiS_3_ system, a 3 × 3 × 3 supercell of the primitive cell was used. The experimental cell parameters obtained by Celasun et al. [24] (and reported in Table S1 in the Supplementary Materials) were used. This system consists of 54 sites, 27 of which are occupied by either a Ti or Li atom (9 Ti and 18 Li). The combinatorial analysis identifies 4023 symmetry-irreducible structures. The identification number (ID) is obtained by the random generation of the structures by the CRYSTAL code.

In order to identify structural indicators to distinguish and classify this large number of structures and rationalize the occurrence of specific geometric arrangements, two geometrical descriptors were introduced: the average Ti-Ti distance $\bar{d_{Ti-Ti}}$ (that is the average of the shortest distances among all Ti-Ti couples) and the standard deviations of such distances $\sigma_{d_{Ti-Ti}}$ as defined in Equations (1) and (2) (N is the number of Ti atoms in the cell)

(1) $$\bar{d_{Ti-Ti}}=\frac{1}{N\left(N-1\right)}\sum_{i=1}^{N}\sum_{j=1}^{N}d_{Ti_{i}-Ti_{j}}$$

(2) $$\sigma_{d_{Ti-Ti}}=\sqrt{\frac{\sum {\left(d_{Ti-Ti}-\bar{d_{Ti-Ti}}\;\right)}^{2}}{N\left(N-1\right)}}$$

**Figure 3:**
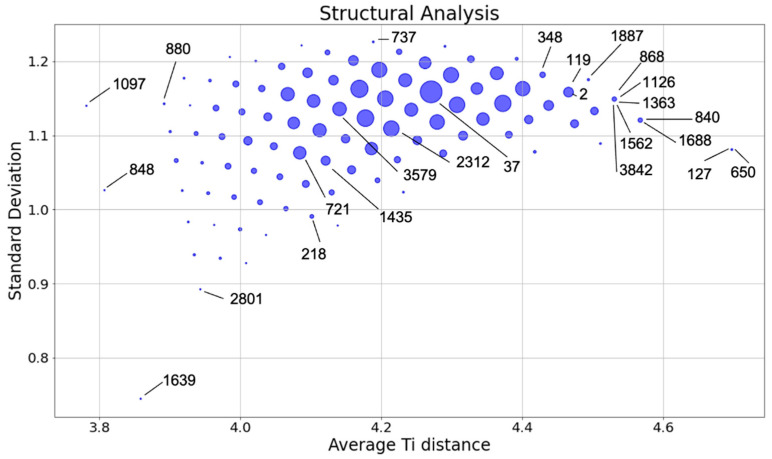
Distribution of the 4023 symmetry irreducible structures of Li_2_TiS_3_ in terms of $\sigma_{d_{Ti-Ti}}\;\mathrm{vs.}\;\bar{d_{Ti-Ti}}$ as specified in Equations (1) and (2). For each group, the size of the marker is proportional to the number of structures belonging to it. The position of the 25 structures selected for this study were labelled in the graph.

The distribution of the 4023 symmetry-irreducible Li_2_TiS_3_ structures in terms of $\sigma_{d_{Ti-Ti}}\;\mathrm{vs.}\;\bar{d_{Ti-Ti}}$ is plotted in Figure 3. By using these criteria, the 4023 structures are grouped in 98 subgroups marked on the graph with a circle of variable size.

The couple $\bar{d_{Ti-Ti}}$ and $\sigma_{d_{Ti-Ti}}$ is a good descriptor to represent the degree of dispersion or dilution of the 9 Ti atoms over the 27 available lattice positions.

Large $\bar{d_{Ti-Ti}}$ is associated with disordered structures (without or with little Ti nanostructuring); small $\bar{d_{Ti-Ti}}$ implies a certain degree of Ti nanostructuring from rows and vicinal Ti rows to Ti planes as $\sigma_{d_{Ti-Ti}}$ increases. The general trend is summarized in in Table 3, where the degree of disorder is highlighted by the darkness of the background (darker areas correspond to more disordered structures).

A representative subset of the 4023 structures consisting of 25 structures (structures selected in this study are highlighted in Figure 3) were selected by combining the information reported in Figure 3 and Table 3. The selected structures cover a representative range of $\bar{d_{Ti-Ti}}$ and $\sigma_{d_{Ti-Ti}}$ values and namely:

- structures 1639, 848, 1097, 127, and 650 were chosen as they are the outlier points
- structures 2801, 880, 737, 218, and 840 represent the borders of the main distribution
- structures 37, 3579, and 2312 were chosen as they sample the center of the main distribution

In addition:

- structures 868, 1126, 1363, 1526, and 3842 were deliberately chosen to have the same values of $\bar{d_{Ti-Ti}}$ and $\sigma_{d_{Ti-Ti}}$ in order to study how the electronic properties vary within the same group
- structures 127 and 650 are of particular interest since, despite belonging to the same group, the structure 650 is largely dispersed, whereas the structure 127 has some level of nanostructuring. This nanostructuring can be described as a spiral structure where each Ti atom has two Ti atoms in its second coordination shell.

The 25 structures discussed above were optimized (at the B3LYP/Basis B level). Their structural and energy data are reported in Tables S2 and S3 of the Supplementary Materials, ordered in terms of decreasing stability with respect to the energy of the monoclinic structure. Analysis of the table highlights that:

1. all structures are less stable than the monoclinic one by 0.1–0.7 eV per Formula Unit (FU);
2. dispersed structures (top of Table S3) result, overall, to be more stable than the structures displaying some level of ordering/nanostructuring (bottom of Table S3). The energy of the dispersed systems varies in a range of 0.1 eV/FU range (from 0.15 eV for structure 1887 to 0.24 eV for structure 840); the energy of the nanostructured systems varies in a range of 0.5 eV/FU (from 0.45 eV for structure 2312 to 0.70 eV for structure 880). The only exception in this trend (among the structures selected for this study) is structure 2, whose energy falls into the dispersed energy ones, despite the presence of a Ti row. However, by looking at its structure, it can be observed that aside from the single Ti row, the other Ti atoms appear to be evenly dispersed. This is also confirmed by the fact that structure 2 can be found at the top right corner of Figure 3 and it can, therefore, be considered to be at the border between the dispersed and nanostructured structures as it is also suggested by its energy and band gap;
3. the band gap follows a similar trend as for relative stability, as it decreases regularly with the increasing level of Ti nanostructuring, from 2.5 eV (dispersed structures) to 1.71 eV (structures with multiple Ti rows or plane). As highlighted before, the hybrid B3LYP method generally overestimate the band gap values, we expect that a similar behavior occurs in these systems;
4. the structure is pseudo-cubic with small deviations from the ideal cubic one; structures showing some ordering tend to have larger volumes that can be seen as an indication of the distortion that nanostructuring imposes to the lattice. In order to further study such distortion, the maximum and minimum Li-S, Li-Ti, and Li-Ti distances are reported in Table S3. The ordered structures have the largest variability among these values. The effect of this distortion on the electronic structure of the material is discussed below.

Hereafter, we referred to “dispersed” and “ordered” to roughly classify structures according to the degree of Ti nanostructuring in the lattice: “dispersed” with no or low (at most one Ti row) Ti clustering; “ordered” with high Ti nanostructing (multiple Ti rows or plane).

An interesting correlation between geometry and electron structure can be observed in Tables S2 and S3 of the Supplementary Materials. To better understand these relationships at the atomistic level, six structures (three from the most stable and three from the less stable ones in the 25 subset) were selected and analyzed further.

**Figure 4:**
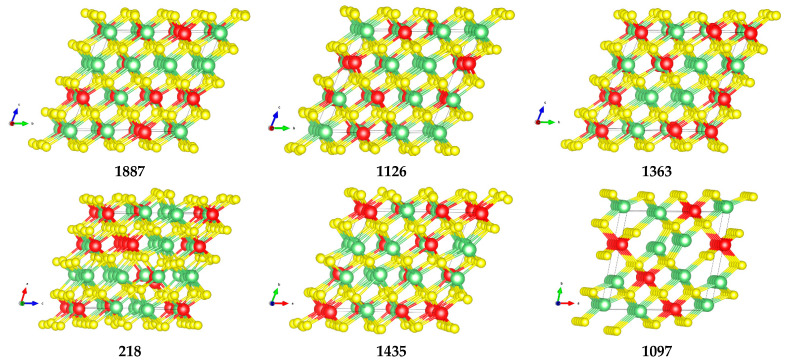
Examples of dispersed and ordered structures: **1887**, **1126**, **1363**, **218**, **1435,** and **1097**. Ti (red), Li (green), S (yellow). The structures are represented in their primitive crystal lattice and in different orientations in order to highlight rows/planes when present. The structures were visualized and the figures were produced with the help of VESTA software [41].

The six structures (1887, 1363, 1126, 218, 1435, 1097) are sketched in Figure 4 and selected properties reported in Table 4.

**Figure 5:**
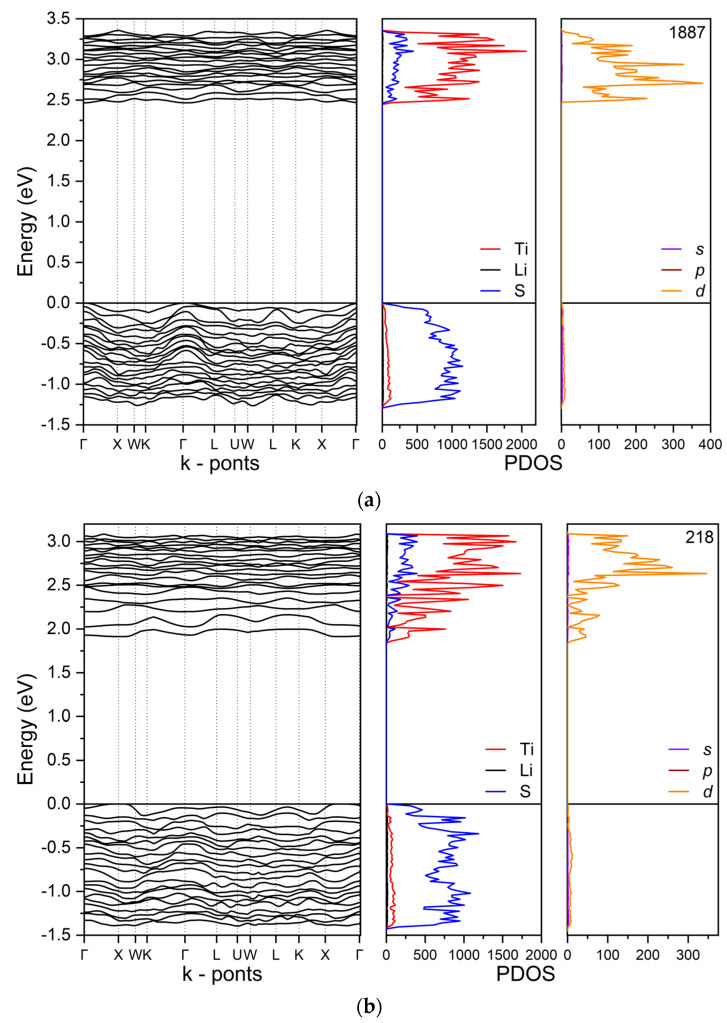
Band Structure and PDOS of pseudocubic “dispersed” structures 1887 (**a**) and “ordered” 218 (**b**). Ti, S, and Li projections are reported in the middle panels of (**a**,**b**). In the right panels 4s, 4p, and 3d projection over a single Ti atom are reported. In the case of structure 218 the Ti atom is one from the Ti row. See also Figure 4 and Table 4.

The band structure and PDOS of the six structures are depicted in Figure S4. In Figure 5, a more detailed description of the band structure and the PDOS of structures 1887 (dispersed) and 218 (nanostructured) is reported. The calculated band gap reported in Table 4 and Figure 5 are strongly influenced by the hybrid B3LYP functional, which overestimate its values. Inspection of the figure shows that the top of the valence band is characterized by 3sp sulfur states, whereas the bottom of the conduction band is mainly due to Ti states derived by 3d (e_2g_) in the distorted octahedral environment of Ti in the pseudo-cubic structures. 1s Li states are well-localized at 55 eV below the VB. Mulliken charges and PDOS indicate a significant covalence in the Ti-S bond, whereas Li-S bonds show a dominant ionic character. Structure 1887 exhibits a band gap of about 2.46 eV that drops to 1.85 eV in structure 218 following the general trend highlighted in Table S2 and also confirmed by the band structures of all the six structures reported in Figure S4. For a better understanding of this phenomenon, the PDOS projected onto s, *p*, d orbitals for a single titanium atom belonging to each structure are also reported in Figure 5. In the case of structure 218, a Ti atom belonging to a Ti row was selected. Analysis of the PDOS shows that the drop of the band gap is due to a downshift of 3d states (e_2g_ states in and ideal octahedral arrangement) related to the Ti atom belonging to the row. This behavior can be correlated to the larger distortion from the ideal Ti cubic environment in structures showing a significant ordered arrangement in the Ti sublattice: deformation imposed by the Ti atoms nanostructuring can be linked to the wide difference between the minimal and maximum values of the Ti-S, Li-S, and Ti-Li distances occurring in the cells, see Table S2 of Supplementary Materials; the result is a reduced d-d splitting due to the less effective ligand field.

**Figure 6:**
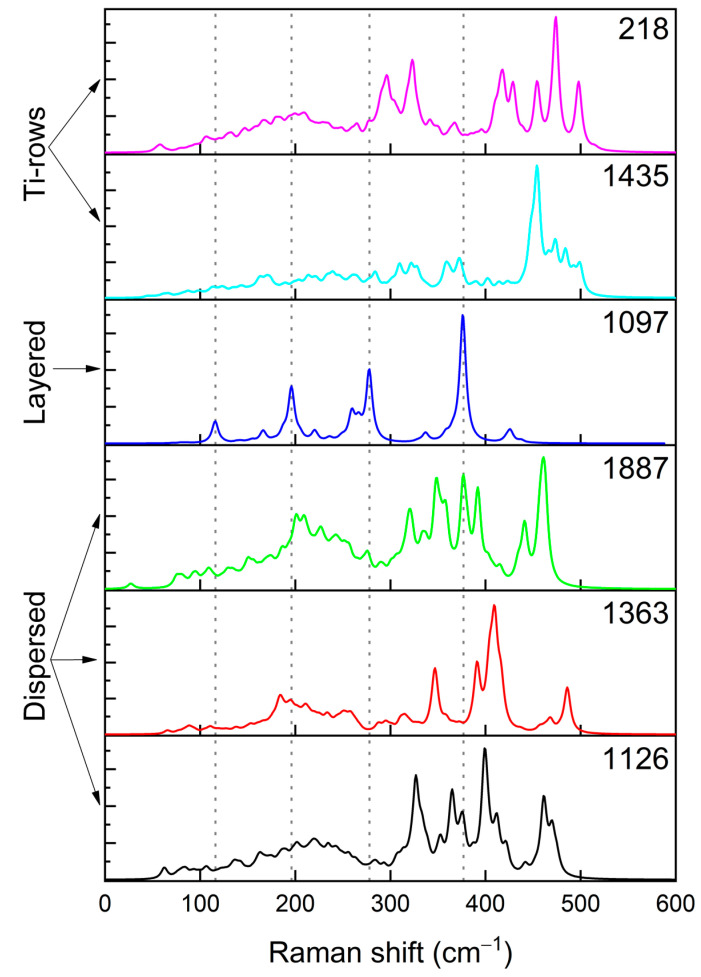
Calculated Raman spectra of the six structures described in Table 4 and Figure 4.

Raman spectra of the six selected structures are reported in Figure 6. Of particular interest is the structure 1097 (characterized by a layered internal organization), whose Raman spectrum is represented through the blue curve in Figure 6. In this spectrum, the highest peak at 369 cm^−1^ and the shoulder at 380 cm^−1^ are associated, respectively, to the Ti-S and Li-S in-phase asymmetric stretching along the direction perpendicular to the titanium planes. The peak falling close to 280 cm^−1^ splits in two parts; these features are assigned to Li-S in-phase stretching, respectively, parallel and perpendicular to the titanium planes. The peak at 193 cm^−1^ can be associated to the Ti-S bond stretching perpendicular to the ordered titanium planes. By studying the animation of modes, we were able to assign the significant peak at lower wavenumbers 115 cm^−1^ to bulk vibrations of the kind of layers twisting. In structures characterized by titanium rows (pink and turquoise curves of Figure 6), the peak at 380 cm^−1^ related to the Ti-S bond stretching is still present. However, it is now due to the Ti-S bond stretching involving Ti atoms localized on the rows with a contribution from the Li-S stretching. The peaks at lower wavelength are associated to Li-S bond stretching nearby the titanium rows, suggesting that the presence of ordered structures influences the vibrational behavior of the surrounding environment. In particular, the common peak at 300 cm^−1^ is associated to the S-Li-S asymmetric stretching when these are close to rows of titanium atoms. Peaks above 400 cm^−1^ can be assigned to in phase Li-S and Ti-S stretching modes, always in connection with Ti rows in nearby.

In “dispersed” structures, in spite of some variability in the spectra, it is possible to identify some common spectroscopic features. The region below 300 cm^−1^ attains to bulk modes mainly due to Li-S stretching along the three different axes. In general, the main peaks, assigned above in “ordered” structures, blue-shift as follows:

- the asymmetric stretching of Ti-S and Li-S bonds are shifted to 400 cm^−1^ by ~30 cm^−1^;
- the symmetric stretching of Li-S bonds is shifted to ~350 cm^−1^, with a Δν of ~50 cm^−1^;
- the symmetric stretching of Ti-S and Li-S bonds falls in the region close to 450 cm^−1^.

Based on the above discussed Raman analysis, this technique proved to be effective to identify the fingerprint linked to the presence of planes and rows in the structure. In fact, the largely ordered structure exhibiting layered structure including Ti atoms are characterized by a few, narrow peaks and a low variability in terms of vibrational modes. By focusing on the region close to 450 cm^−1^, it can be inferred that in structures characterized by the presence of Ti atom rows, a certain number of peaks are present at values higher than 450 cm^−1^ as opposite to the dispersed structures.

Further analysis of the Raman features of pseudo-cubic structures in comparison with the monoclinic one, shows that the main peaks characterizing the monoclinic arrangement (the peaks at 331, 259 cm^−1^) are absent in the cubic structures irrespective to their degree of Ti nanostructuring; see Figure S6 of Supplementary Materials for a comparison between Raman spectra of the pseudocubic dispersed structure 1363 and the monoclinic form of Li_2_TiS_3_). In comparing the spectra, it appears evident that the two iso-composites can be easily distinguished by Raman spectroscopy.

**Figure 7:**
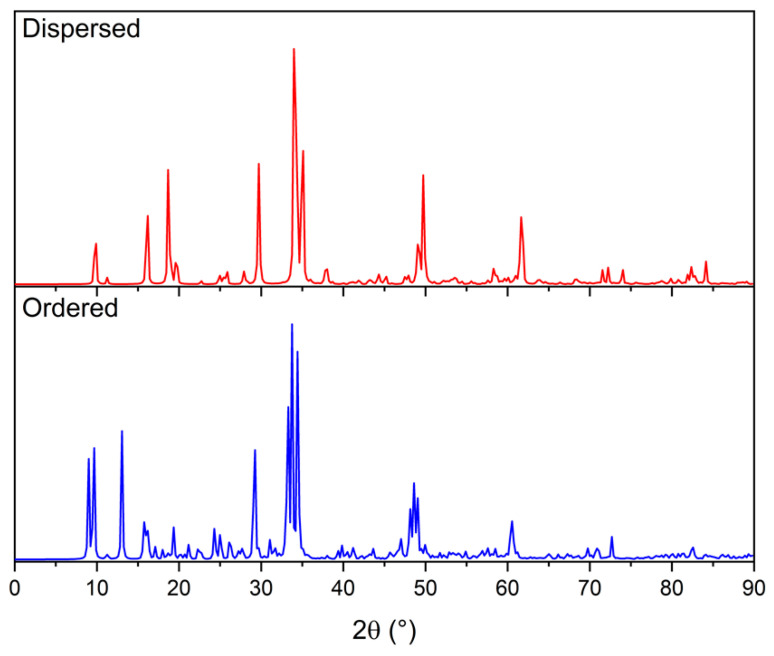
Calculated XRD pattern for dispersed (red) and ordered (blue) structure.

Finally, it can be observed that XRD patterns can discriminate, as expected, between cubic and monoclinic Li_2_TiS_3_, but they are also sensitive to the local arrangement inside different pseudocubic structures. This finding is highlighted in Figure 7 that reports the XRD pattern of a dispersed and a row-like cubic Li_2_TiS_3_. The two patterns show some common features (the highest peak at 35° is a common feature between all the structures) that are also shared by the other pseudo cubic Li_2_TiS_3_ (see Figure S3 of Supplementary Materials). However, in the dispersed structure the peaks at high (50°, 63°, 75°) and low (10°, 30°) angles are split in two, features that are indeed peculiar of this kind of internal organization.

## 4. Conclusions

In this work, disorder cubic Li_2_TiS_3_ was investigated by means of periodic DFT calculations. This material has attracted the attention of the scientific community because of its promising features: high energy capacity, no use of critical raw materials, and low cost.

Performances of the hybrid B3LYP functional combined with a TZV Gaussian basis set for the accurate prediction of the electronic, XRD and Raman properties of Li-Ti sulfides were tested on well-known compounds by comparing simulated results with the data available in the literature. Precursors and systems based on lithium, sulfur, and titanium were selected for their similarity with the system of interest, such as Li_2_S, TiS_2_, TiS_3_, LiTiS_2_, and monoclinic Li_2_TiS_3_.

Pseudo-cubic Li_2_TiS_3_ structures were constructed starting from experimental, and a 3 × 3 × 3 supercell was used in order to obtain the correct stoichiometry. Following this approach, systems consisting of 54 atoms were obtained: 27 sulfur, 18 lithium, and 9 titanium. With the help of a procedure developed by some of us for solid solutions, we were able to reduce all the possible configurations (~4.7 millions) to the symmetry-irreducible ones, hence 4023 structures had to be further analyzed. A rationalization of all the possible configurations was carried out by using two geometry descriptors, the average Ti-Ti distance, and the corresponding standard deviation. Therefore, 98 subgroups characterized by different distributions of Ti atoms into the Li sub-lattice were obtained. These subgroups were divided further into two macro groups on the basis of the local arrangement of the Ti atoms; we referred to each as “dispersed” and “ordered” structures, characterized respectively by no or low (one Ti row) and by high (multiple Ti rows or Ti planes) degree of Ti nanostructuring. Between them, 25 structures were selected in order to build a subset as representative as possible of the variety of lattice arrangements present in the whole ensemble. The 25 pseudocubic structures were found to be less stable than the monoclinic Li_2_TiS_3_ one. In addition, stability and band gap values were found to increase regularly with the increase of Ti dispersion in the lattice. The trend was rationalized by observing that the Ti nanostructuring destabilizes the lattice since it imposes larger deformations from the ideal cubic Ti environment; that finding was also confirmed by a larger cell volume and a larger variability in the Li-S, Ti-S, and Li-Ti distances in the “ordered” structures. The less effective octahedral ligand field experienced by Ti atoms, reduces the d-d splitting and thus the gap.

Raman spectra were found to be an effective tool to discriminate not only between pseudocubic and monoclinic Li_2_TiS_3,_ but also between “dispersed” and “ordered” structures since each peculiar Ti geometry motif produces specific peaks in the Raman spectrum that proved, indeed, to be a valuable tool for the structural recognition of Li_2_TiS_3_ isocomposites.

## Figures and Tables

**Table 2:** Raman data. Simulated Raman peaks are compared with the data available in the literature.

	Set A	Set B	Experimental
**Li_2_S**			
Raman Peak	372 cm^−1^	375 cm^−1^	370 cm^−1^ [38]
**TiS_2_**			
Raman Peak 1	248 cm^−1^	244 cm^−1^	250 cm^−1^
Raman Peak 2	359 cm^−1^	349 cm^−1^	334 cm^−1^ [39]
**TiS_3_**			
Raman Peak 1	304 cm^−1^	298 cm^−1^	299 cm^−1^
Raman Peak 2	370 cm^−1^	364 cm^−1^	365 cm^−1^
Raman Peak 3	561 cm^−1^	565 cm^−1^	560 cm^−1^ [37,40]

**Table 3:** Summary of the properties corresponding to different areas of the Figure 3. The darkness of the background corresponds to the level of disorder (darker areas correspond to more disordered structures).

	$\mathrm{Small}\;\bar{d_{Ti-Ti}}$	$\mathrm{Large}\;\bar{d_{Ti-Ti}}$
Large $\sigma_{d_{Ti-Ti}}$	Nanostructuring (Ti planes)	Disordered structures (no Ti nanostructuring)
Small $\sigma_{d_{Ti-Ti}}$	Ordered structures (Ti clusters, vicinal rows)	Disordered structures (local Ti nanostructuring)

**Table 4:** Energetic, electronic, and structural data for six selected structures. **ΔE** is the relative stability (in eV/FU) of the pseudocubic structures with respect to the monoclinic one; V1 is the volume of the primitive cell; q is the gross Mulliken charge in |e|. See also Figure 4.

Structure ID	ΔE (eV)	Band Gap (eV)	V1 (Å^3^)	q (Ti)	q (Li)	q (S)	Description
**1887**	0.146	2.465	948.99	0.64	0.85	−0.78	Dispersed
**1363**	0.148	2.531	950.81	0.63	0.85	−0.78	Dispersed
**1126**	0.164	2.509	953.42	0.64	0.85	−0.78	Dispersed
**218**	0.589	1.854	1002.55	0.57	0.85	−0.76	Ti rows
**1097**	0.695	1.795	1046.59	0.57	0.85	−0.76	Ti Planes (1 0 0)
**1435**	0.699	1.706	1030.19	0.56	0.85	−0.75	Ti rows

## Supplementary Materials

The following supporting information can be downloaded at: https://www.mdpi.com/article/10.3390/nano12111832/s1, Figure S1. Electronic band structure and density of states of: (a) Li_2_S, (b) monoclinic Li_2_TiS_3_, (c) LiTiS_2_, (d) TiS_2_ and (e) TiS_3_; Figure S2. XRD patterns of (a) Li_2_S, (b) monoclinic Li_2_TiS_3_, (c) LiTiS_2_, (d) TiS_2_ and (e) TiS_3_; Figure S3. XRD pattern for selected disordered cubic Li_2_TiS_3_; Figure S4. Band structures and Density of States for disordered structures: 1126 (a), 218 (b), 1363 (c), 1435 (d), 1887 (e), 1097 (f); Figure S5. Density of States for disordered structures: 1126 (a), 218 (b), 1363 (c), 1435 (d), 1887 (e), 1097 (f). Focus on the atom’s projections; Figure S6. Comparison between disordered pseudocubic structure 1363 and monoclinic Li_2_TiS_3_; Table S1. Crystallographic data for disordered cubic Li_2_TiS_3_. Space group Fm-3m a=b=c= 5.0855 Å; Table S2. Structural and electronic properties of the selected structures: focus on the stability, band gap and distances between atoms. The ΔE reported in the table is the energy difference within respect to the Monoclinic structure, normalized per Formula Units; Table S3. Structural and electronic properties of the selected structures: focus on the stability, band gap, distances between atoms.
