# Supplementary material for: Disordered Rock-Salt Type Li2TiS3 as Novel Cathode for LIBs: A Computational Point of View
Source: Nanomaterials (Basel). 2022 May 27;12(11):1832. doi: 10.3390/nano12111832 (PMC9181842; doi:10.3390/nano12111832)
Supplement: Supplementary file 1 [file nanomaterials-12-01832-s001.zip › nanomaterials-1737271-supplementary.pdf]

Disordered Rock-Salt Type Li_2TiS_3 as Novel Cathode for LIBs: A Computational Point of View

Riccardo Rocca [1,2,*], Mauro Francesco Sgroi [2], Bruno Camino [3], Maddalena D'Amore [1] and Anna Maria Ferrari [1,*]

[1] Department of Chemistry and NIS, University of Turin, 10125 Torino, Italy; maddalena.damore@unito.it
[2] Centro Ricerche FIAT S.C.p.A, 10043 Orbassano, Italy; mauro.sgroi@crf.it
[3] Department of Chemistry, Imperial College, London SW7 2AZ, UK; b.camino13@imperial.ac.uk
* Correspondence: riccardo.rocca@unito.it or riccardo.rocca@external.crf.it (R.R.); anna.ferrari@unito.it (A.M.F.)

1. Precursors

1.1. Electronic Band Structure and Density of States

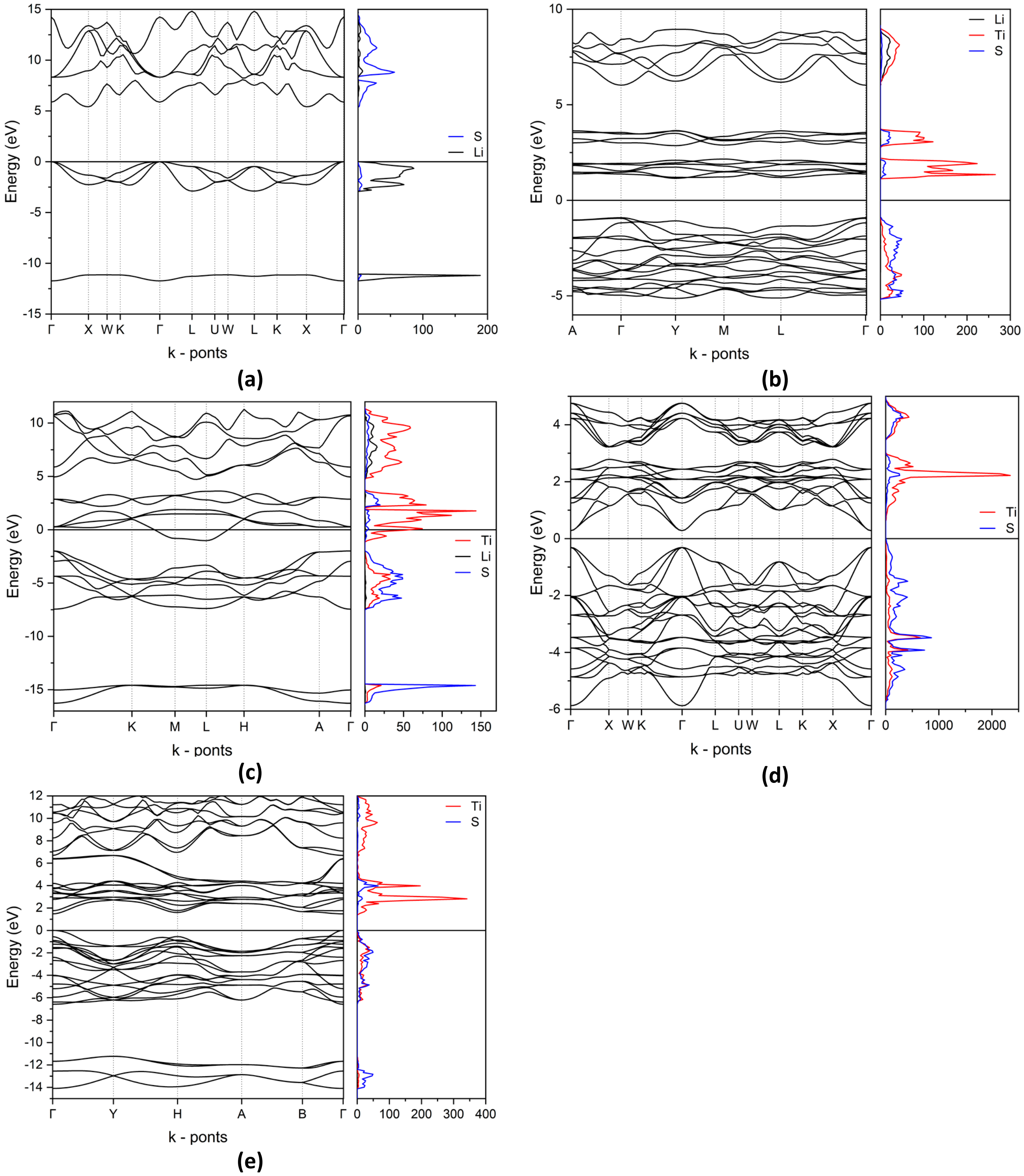

Figure S1. Electronic band structure and density of states of: a) Li_2S, b) monoclinic Li_2TiS_3, c) $LiTiS_2$, d) TiS_2 and e) TiS_3.

1.2. XRD pattern

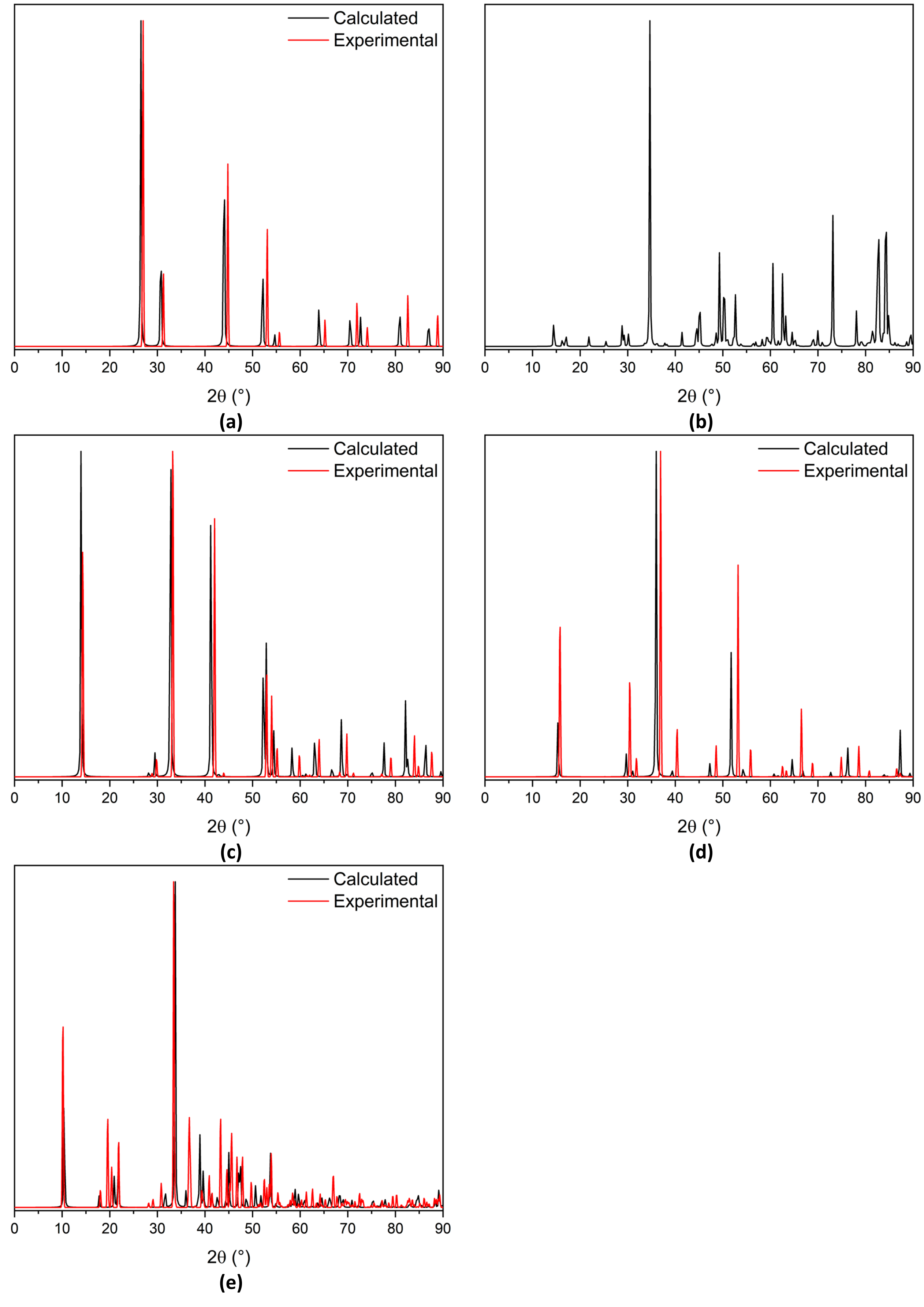

Figure S2. Simulated and experimental XRD patterns of a) Li_2S, b) monoclinic Li_2TiS_3, c) $LiTiS_2$, d) TiS_2 and e) TiS_3.

2. Disordered Structures

2.1. XRD pattern

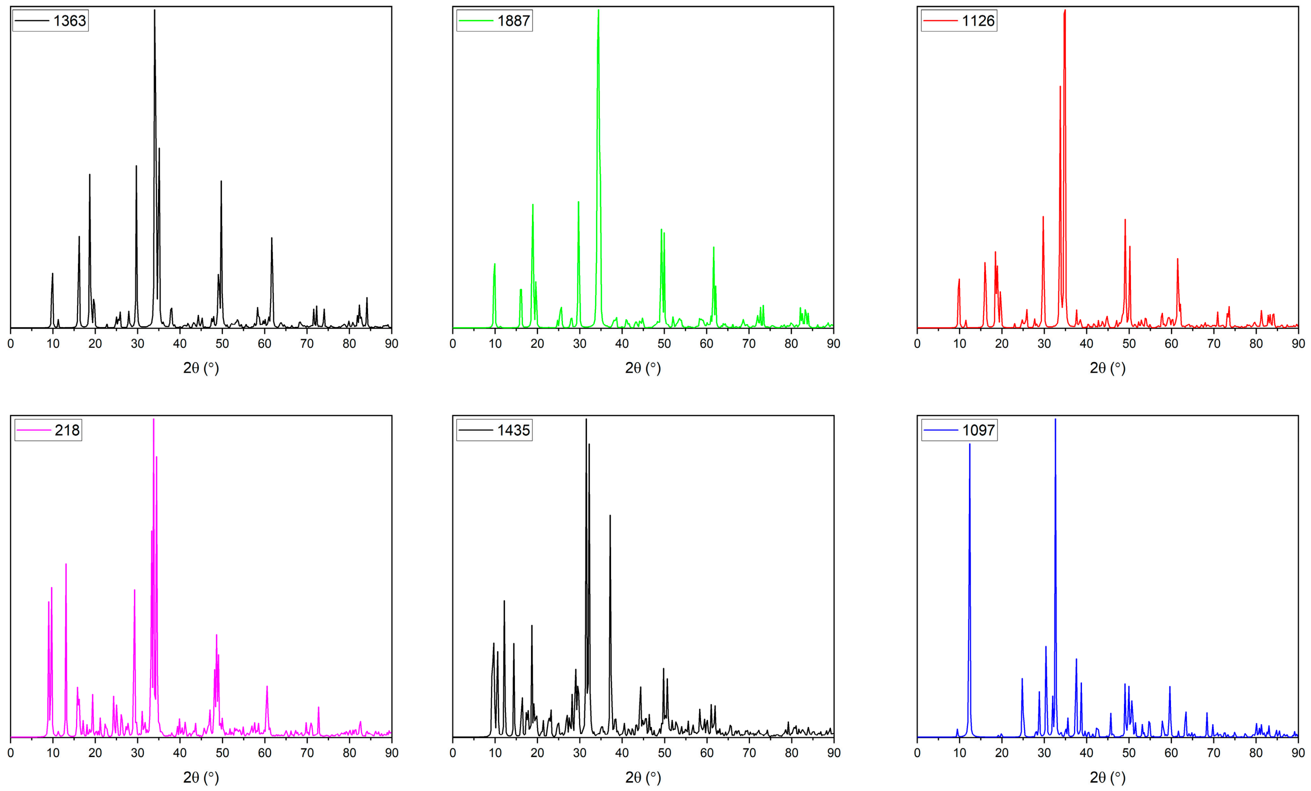

Figure S3. Computed XRD pattern for selected disordered cubic Li_2TiS_3.

2.2. Electronic Band Structure and Density of States

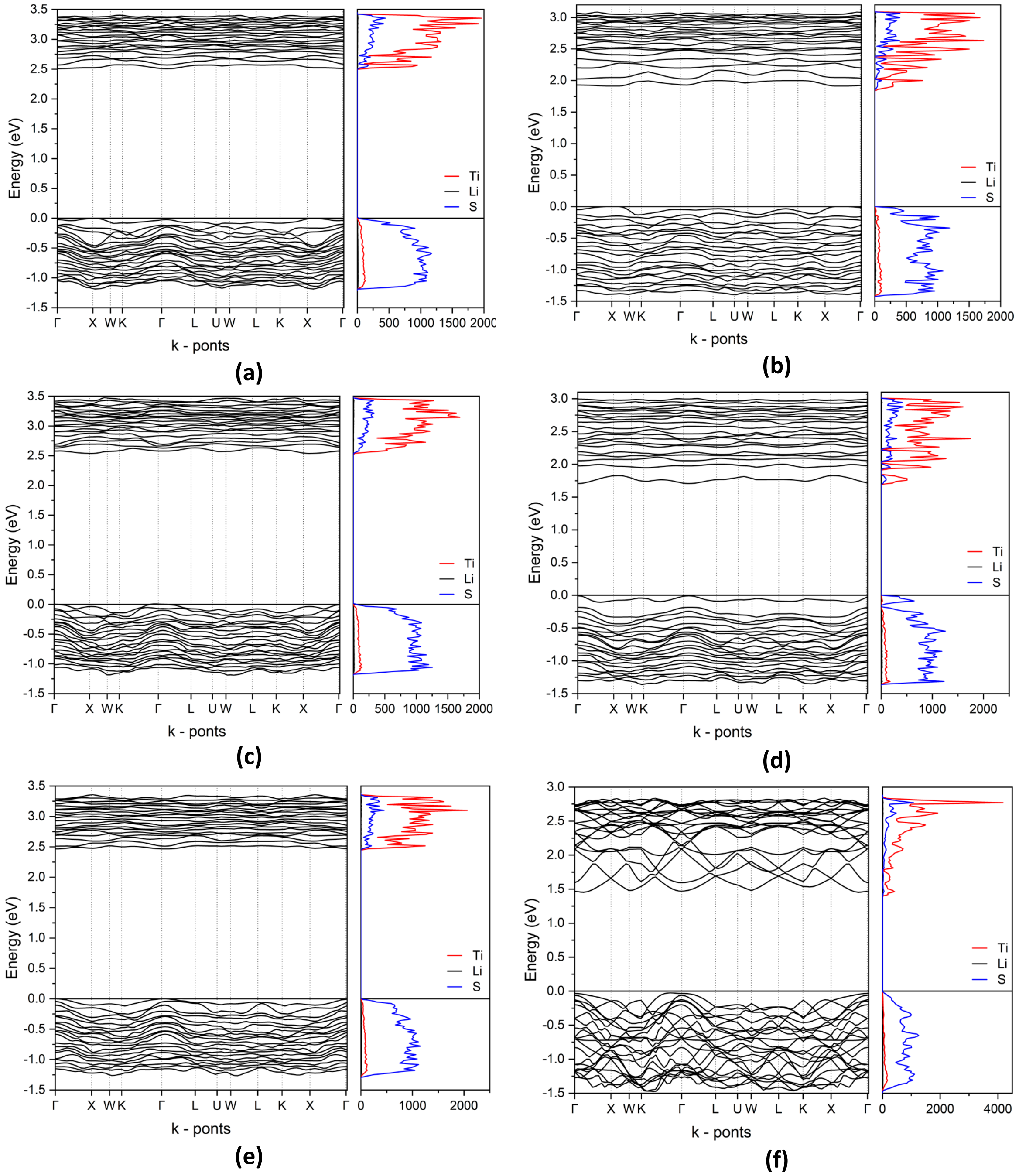

Figure S4. Band structures and Density of States for disordered structures: 1126 (a), 218 (b), 1363 (c), 1435 (d), 1887 (e), 1097 (f)

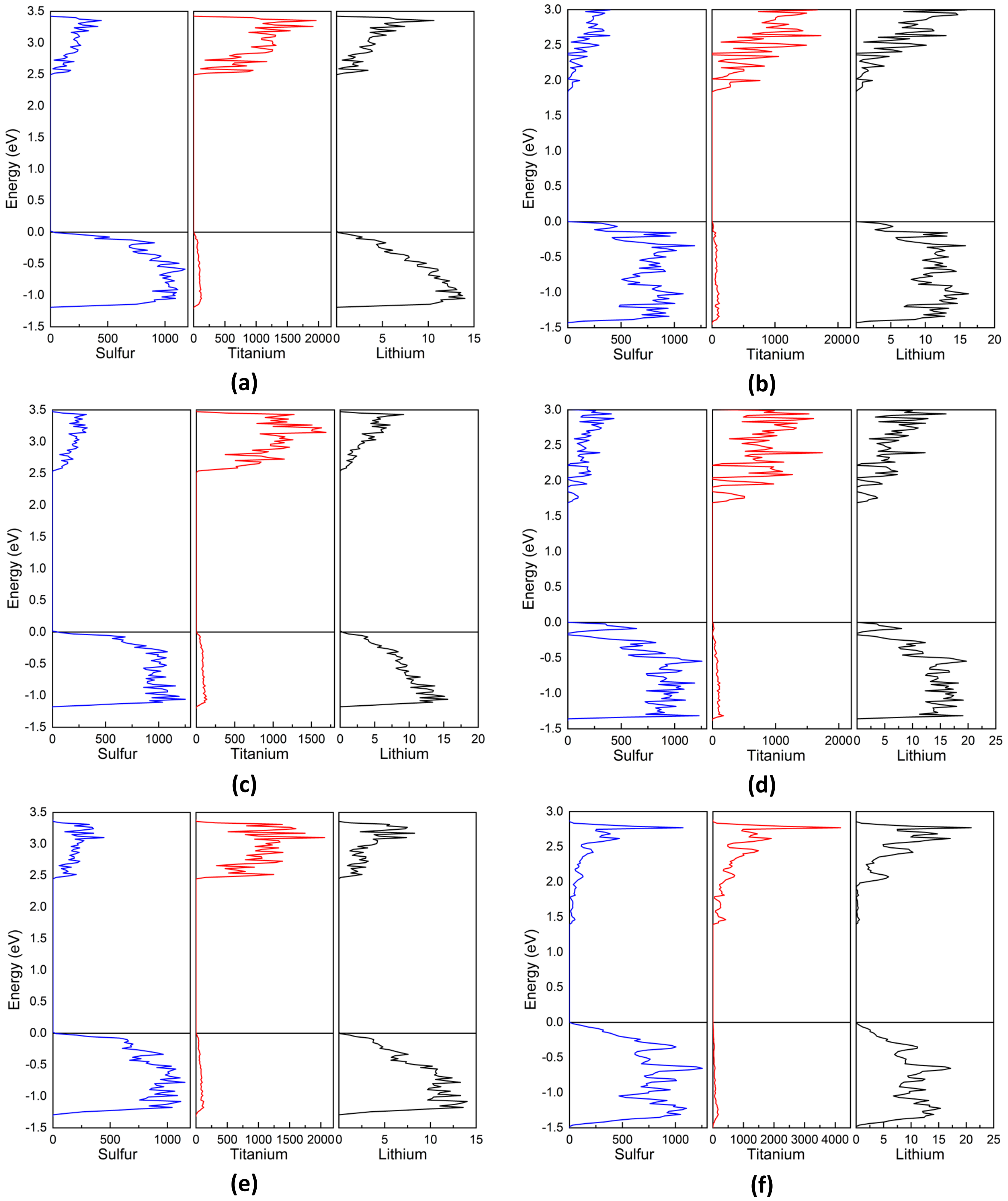

Figure S5. Density of States for disordered structures: 1126 (a), 218 (b), 1363 (c), 1435 (d), 1887 (e), 1097 (f). Focus on the atom's projections.

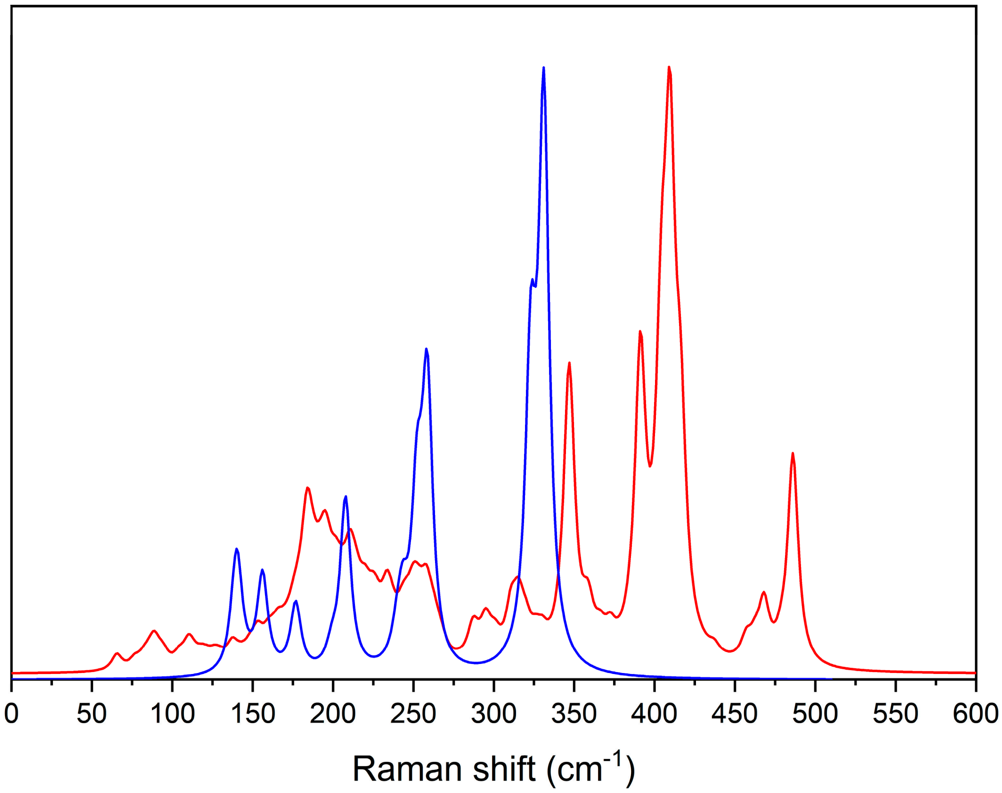

Figure S6. Comparison between Raman responses of disordered pseudocubic structure 1363 (red) and monoclinic Li_2TiS_3 (blue).

Table S1. Crystallographic data for disordered cubic Li_2TiS_3. Space group Fm-3m a=b=c= 5.0855 Å.

Site	WP	x	y	z	Atom	sof	Biso
**Li**	4a	0	0	0	Li^+	0.6644	1.34
**Ti**	4a	0	0	0	Ti^{4+}	0.3385	
**S**	4b	1/2	1/2	1/2	S^{2-}	1.00	

Table S2. Structural and electronic properties of the selected structures: focus on the stability, band gap and distances between atoms. The ΔE reported in the table is the energy difference within respect to the Monoclinic structure, normalized per Formula Units.

Structure ID	Ti-S min (Å)	Ti-S max (Å)	Li-S min (Å)	Li-S max (Å)	Ti-Li min (Å)	Ti-Li max (Å)	ΔE (eV)	Band Gap (eV)	Multiplicity	Description
**1887**	2.227	3.302	2.492	3.000	3.298	3.785	0.146	2.465	648	Dispersed
**1363**	2.201	3.167	2.523	2.937	3.286	3.759	0.148	2.531	348	Dispersed
**1126**	2.210	3.400	2.517	3.128	3.290	3.796	0.164	2.509	1296	Dispersed
**3842**	2.197	3.409	2.500	3.011	3.291	3.797	0.174	2.549	1296	Dispersed
**348**	2.209	3.425	2.483	3.026	3.264	3.773	0.177	2.476	1296	Dispersed
**650**	2.208	3.429	2.482	3.032	3.264	3.771	0.177	2.476	1296	Dispersed
**1526**	2.208	3.427	2.483	3.029	3.264	3.771	0.177	2.476	18	Dispersed
**3579**	2.208	3.430	2.484	3.033	3.264	3.766	0.177	2.478	1296	Dispersed
**868**	2.220	3.222	2.492	3.110	3.325	3.814	0.182	2.199	324	Dispersed
**119**	2.212	3.284	2.506	3.032	3.299	3.776	0.189	2.513	1296	Dispersed
**2**	2.203	3.122	2.477	2.961	3.268	3.791	0.206	2.163	1296	Ti rows
**1688**	2.213	3.216	2.420	2.946	3.266	3.809	0.236	2.296	1296	Dispersed
**840**	2.208	3.552	2.469	3.158	3.279	3.795	0.240	2.417	1296	Dispersed
**37**	2.542	2.542	2.542	2.542	3.595	3.595	0.269	1.957	1296	Ti rows
**2312**	2.195	4.032	2.322	3.656	2.845	4.058	0.454	1.720	1296	Ti rows
**127**	2.200	3.362	2.301	3.406	3.232	3.953	0.496	1.500	72	Ti spiral
**218**	2.169	4.183	2.294	3.749	2.957	3.881	0.589	1.854	648	Ti rows
**721**	2.174	3.650	2.302	3.556	3.309	3.892	0.628	1.405	1296	Ti rows
**737**	2.151	3.994	2.290	3.983	3.300	4.225	0.695	1.794	9	Ti rows
**848**	2.152	3.999	2.289	3.980	3.304	4.230	0.695	1.794	108	Ti rows
**1097**	2.152	4.002	2.289	3.977	3.304	4.230	0.695	1.795	1296	Ti Planes (1 0 0)
**1639**	2.243	2.978	2.459	3.385	3.305	3.881	0.695	1.428	12	Ti Planes (1 1 1)
**880**	2.151	3.995	2.289	3.982	3.302	4.225	0.696	1.794	648	Vicinal Ti rows
**2801**	2.152	4.002	2.289	3.977	3.302	4.230	0.696	1.798	648	Ti rows
**1435**	2.182	3.948	2.320	3.981	2.964	4.128	0.699	1.706	1296	Ti rows

Table S3. Structural and electronic properties of the selected structures: focus on the stability, band gap and distances between atoms. V 1 refers to the primitive cell, while V 2 is referred to the conventional cell.

**Structure ID**	***a*** **(Å)**	***b*** **(Å)**	***c*** **(Å)**	**α (Å)**	**β (Å)**	**γ (Å)**	**V 1 (Å³)**	**V 2 (Å³)**
**1887**	15.599	15.736	15.466	89.7	90.4	89.8	948.99	3795.97
**1363**	15.799	15.361	15.672	90.0	90.2	90.2	950.81	3803.22
**1126**	15.496	15.437	15.944	89.8	89.6	90.4	953.42	3813.66
**3842**	15.408	15.561	15.922	90.4	90.9	90.8	954.16	3816.64
**348**	15.523	15.808	15.562	89.4	89.9	90.6	954.55	3818.19
**650**	15.525	15.813	15.556	89.4	89.9	90.6	954.60	3818.41
**1526**	15.524	15.811	15.555	89.4	89.9	90.6	954.41	3817.63
**3579**	15.526	15.818	15.548	89.4	89.9	90.6	954.46	3817.82
**868**	15.463	15.400	15.997	90.3	90.0	90.0	952.32	3809.28
**119**	15.640	15.496	15.706	89.9	89.7	89.9	951.58	3806.33
**2**	15.583	15.422	15.763	89.7	90.2	90.3	947.03	3788.10
**1688**	15.721	15.601	15.501	90.6	90.4	90.5	950.35	3801.39
**840**	16.099	15.319	15.532	89.4	89.3	91.0	957.37	3829.48
**37**	15.300	15.727	15.891	88.6	90.3	88.5	955.43	3821.75
**2312**	13.979	16.081	18.232	101.2	88.3	84.7	999.69	3998.74
**127**	13.520	15.979	18.365	100.8	86.7	84.1	965.94	3863.77
**218**	18.777	13.711	16.045	82.3	100.8	87.4	1002.55	4010.20
**721**	15.234	16.357	15.550	90.0	90.0	91.7	968.29	3873.16
**737**	14.117	16.978	17.476	89.2	90.2	90.9	1046.90	4187.59
**848**	14.117	16.993	17.454	89.2	90.2	90.9	1046.50	4185.99
**1097**	14.114	17.002	17.450	89.2	90.2	90.9	1046.59	4186.37
**1639**	14.376	16.520	16.519	95.3	90.0	90.0	976.60	3906.40
**880**	14.118	16.978	17.469	89.2	90.2	90.9	1046.56	4186.25
**2801**	14.106	17.010	17.460	89.2	90.2	90.9	1047.08	4188.31
**1435**	17.060	14.500	16.693	91.6	86.7	90.7	1030.19	4120.78

In the following we report the CIF files for selected structures: 1887, 1363, 1126, 1435, 218, 1097.

1887
_chemical_formula_sum 'Li18 S27 Ti9'
_chemical_formula_moiety
;
Li18 S27 Ti9
;
loop_
_cell_volume 948.9931
_symmetry_space_group_name_H-M 'P 1'
_symmetry_cell_setting triclinic
_symmetry_Int_Tables_number 1
loop_
_symmetry_equiv_pos_site_id
_symmetry_equiv_pos_as_xyz
1 x,y,z
_cell_length_a 11.0629114
_cell_length_b 10.9433021
_cell_length_c 11.1001638
_cell_angle_alpha 59.867922
_cell_angle_beta 59.647456
_cell_angle_gamma 60.320916
_cell_formula_units_Z 1
loop_
_atom_type_symbol
_atom_type_radius_bond
Li 1.23
S 1.04
Ti 1.4
loop_
_atom_site_label
_atom_site_type_symbol
_atom_site_fract_x
_atom_site_fract_y
_atom_site_fract_z
Li1 Li 0.995209215791944 8.06574621491033E-03 0.991007507303825
Li2 Li 7.21201701994103E-04 1.49470920766545E-03 0.663615458644501
Li3 Li 0.998704025570171 0.335931479190899 4.00741036368266E-03
Li4 Li 0.994526461192823 0.3482867264626 0.340574326522195
Li5 Li 0.989190552407253 0.3461950599171 0.666929474157696
Li6 Li 5.69555473811951E-03 0.6542903828346 0.681021588322896
Li7 Li 0.328204468353999 0.999998615233596 0.652856583515002
Li8 Li 0.3339320919813 0.336575343948999 2.80803284485709E-03
Li9 Li 0.3335382610473 0.329386126883299 0.335550842583001
Li10 Li 0.3280634014748 0.670401595667 0.996500541948752
Li11 Li 0.330546503140999 0.6593499980015 0.346243775057601
Li12 Li 0.32598546047 0.6704816813513 0.668200423827602
Li13 Li 0.663502397735 9.14930756720944E-03 0.999221375796145
Li14 Li 0.6542274852217 5.62429905256842E-03 0.323314468275201
Li15 Li 0.672173535513099 0.995477235735716 0.665573687384205
Li16 Li 0.6754111093122 0.343326556663299 0.667697426407199
Li17 Li 0.6605850090513 0.6668911417329 0.346181991096597
Li18 Li 0.6732299478654 0.6609026824028 0.671785651770803
S1 S 0.1838778607508 0.137956201504799 0.166957372145201
S2 S 0.1689951182991 0.1656662064956 0.4873691178323
S3 S 0.1978178270966 0.154556476716099 0.834956797582802
S4 S 0.1441566782469 0.5260594142364 0.1672509833954
S5 S 0.180470810471199 0.4781777352304 0.522250443367501
S6 S 0.160817436464599 0.5033413298358 0.853288652474804

S7 S 0.150185486162899 0.8327839008674 0.177911893624701
S8 S 0.1641393367156 0.8527111753334 0.481290640336302
S9 S 0.1556128044748 0.8348250776774 0.834912686275601
S10 S 0.4959209753246 0.174811103491399 0.162434486239101
S11 S 0.510554195832 0.168702912249499 0.495186661662101
S12 S 0.4954883066872 0.1687294805965 0.828034176710402
S13 S 0.5251275269945 0.4812811373968 0.166661351389901
S14 S 0.5064069326793 0.4697252968492 0.5086366120857
S15 S 0.5035310823143 0.500288889358699 0.8393273333898
S16 S 0.4904880374861 0.834321189320499 0.153163087976701
S17 S 0.4806440736998 0.846421927199899 0.483451523064599
S18 S 0.5047130698891 0.8261213888398 0.854038170037098
S19 S 0.8361425075606 0.163988360252899 0.185429511442701
S20 S 0.8422478800677 0.163343911804999 0.481244808595398
S21 S 0.8224273962016 0.184870955126399 0.844788999450002
S22 S 0.808596201993799 0.5061714119279 0.177926717543501
S23 S 0.8396206159324 0.519155910273299 0.492540195677504
S24 S 0.811849991326599 0.4980364408589 0.859571139846504
S25 S 0.835281103837002 0.825786568839099 0.163166297633295
S26 S 0.856255798759897 0.826050907340199 0.486739627195702
S27 S 0.833909439148303 0.8083225942308 0.8604301165613
Ti1 Ti 8.38465539180861E-03 0.979441920337499 0.329345839572701
Ti2 Ti 0.02747227318685 0.675537773219299 0.972090894257144
Ti3 Ti 0.967419665048923 0.6828038243222 0.3157497801521
Ti4 Ti 0.3606155211749 1.40461433211295E-02 0.977735393225658
Ti5 Ti 0.3187257539346 1.13744202039295E-02 0.3551158496155
Ti6 Ti 0.3497880701119 0.3177922631334 0.637814172197103
Ti7 Ti 0.646416560809399 0.3435411782187 0.978740482314478
Ti8 Ti 0.6906334364972 0.3210073159011 0.336730917037501
Ti9 Ti 0.661818886854 0.660418569417699 4.62670227408748E-03
#END

1363
_chemical_formula_sum 'Li18 S27 Ti9'
_chemical_formula_moiety
;
Li18 S27 Ti9
;
loop_
_cell_volume 950.8054
_symmetry_space_group_name_H-M 'P 1'
_symmetry_cell_setting triclinic
_symmetry_Int_Tables_number 1
loop_
_symmetry_equiv_pos_site_id
_symmetry_equiv_pos_as_xyz
1 x,y,z
_cell_length_a 10.9739176
_cell_length_b 11.1096398
_cell_length_c 10.9961298
_cell_angle_alpha 59.504235
_cell_angle_beta 60.958478
_cell_angle_gamma 59.980413
_cell_formula_units_Z 1
loop_
_atom_type_symbol
_atom_type_radius_bond
Li 1.23
S 1.04
Ti 1.4

```
loop_
_atom_site_label
_atom_site_type_symbol
_atom_site_fract_x
_atom_site_fract_y
_atom_site_fract_z
Li1   Li    0.999789689054693   0.999520190697636   0.667067779722099
Li2   Li    0.995614100392381   0.3339340402183   2.47342647139301E-03
Li3   Li    0.995486254315182   0.336131691588699   0.337764993463701
Li4   Li    1.33775983670201E-02   0.646869405828999   0.337230684927597
Li5   Li    5.65548619797579E-03   0.6562314085769   0.670575860291994
Li6   Li    0.3350665564417   0.994987976403126   0.998099888488171
Li7   Li    0.341893165719199   0.993878423024777   0.660443660390594
Li8   Li    0.330097918478   0.3266948604846   0.337289025899397
Li9   Li    0.3308899179798   0.657371497467799   5.56923494558915E-03
Li10   Li    0.341824503503199   0.6691076464436   0.319607256294097
Li11   Li    0.3352835405795   0.667027775527699   0.659428327126193
Li12   Li    0.6564728609267   2.84845980388761E-03   6.26103095154819E-04
Li13   Li    0.651446260635   1.04557803877396E-02   0.332490599237298
Li14   Li    0.670040546825   0.341329419722299   2.30197799120151E-03
Li15   Li    0.6599973985289   0.3304471779159   0.336782854439797
Li16   Li    0.6696980912448   0.331923436589   0.669391811135698
Li17   Li    0.6635341672115   0.6705692869652   0.333426864228494
Li18   Li    0.6729201345763   0.6614969554414   0.663265866696699
S1   S    0.1590187932805   0.154314977644899   0.181971074187498
S2   S    0.1686904036067   0.158198211566   0.499950796662997
S3   S    0.156978027762501   0.154772854742599   0.833981810785498
S4   S    0.1601691893482   0.4988215624912   0.144502108970297
S5   S    0.174070457024   0.465016779686699   0.524732966015798
S6   S    0.1844159715795   0.4794493121509   0.829856506687596
S7   S    0.1454220085219   0.8446830443997   0.173149604041697
S8   S    0.1702336919865   0.8445307730558   0.479479041823293
S9   S    0.143908692665   0.831847672982399   0.854662591167699
S10   S    0.4683431732939   0.170783951523999   0.166916841001698
S11   S    0.5026210870479   0.1596310289486   0.527528287628097
S12   S    0.4810750499846   0.182502658467899   0.837971412917699
S13   S    0.5156225116608   0.5154567122847   0.150589910884197
S14   S    0.498288465020599   0.4929332146575   0.5027539332935
S15   S    0.4932098719261   0.4968328656108   0.8398962717147
S16   S    0.5018898489916   0.8336291412393   0.160353141741197
S17   S    0.4875282178995   0.844857455872099   0.486678539936801
S18   S    0.5272497526939   0.835530919187399   0.825283867808394
S19   S    0.8470496838325   0.144835930452   0.166885278603098
S20   S    0.8349980754279   0.1437895662212   0.499717452483302
S21   S    0.8449056794533   0.1679725478445   0.838373778598801
S22   S    0.837587943672099   0.5248494476449   0.139865360160297
S23   S    0.8469043839187   0.484556533370899   0.512571057513496
S24   S    0.843001975460799   0.493888550049099   0.826962995622297
S25   S    0.841675321877601   0.8335175089482   0.158280512926199
S26   S    0.836381096966902   0.841078427493299   0.4964678072726
S27   S    0.828801377174395   0.8258083962949   0.8373505436774
Ti1   Ti    7.69245692261432E-03   1.21419926029296E-02   0.990061853094524
Ti2   Ti    1.50167813067203E-02   6.10070709459757E-03   0.319926045970897
Ti3   Ti    0.97053926759158   0.3543876855552   0.6854167161729
Ti4   Ti    0.980089453457853   0.690682269829   0.018321912064946
Ti5   Ti    0.3130144565741   1.08850072560496E-02   0.365583038459897
Ti6   Ti    0.3524066283371   0.356933640238399   0.973128491581874
Ti7   Ti    0.3592972793101   0.351115546540299   0.648397678461297
Ti8   Ti    0.6953224301622   0.977797544984579   0.642357225153099
Ti9   Ti    0.6374923032889   0.685040127972299   2.62373300665379E-02
```

#END

1126
_chemical_formula_sum 'Li18 S27 Ti9'
_chemical_formula_moiety
;
Li18 S27 Ti9
;
loop_
_cell_volume 953.4151
_symmetry_space_group_name_H-M 'P 1'
_symmetry_cell_setting triclinic
_symmetry_Int_Tables_number 1
loop_
_symmetry_equiv_pos_site_id
_symmetry_equiv_pos_as_xyz
1 x,y,z
_cell_length_a 11.1190362
_cell_length_b 11.1513532
_cell_length_c 10.9026085
_cell_angle_alpha 60.266069
_cell_angle_beta 60.41905
_cell_angle_gamma 59.018987
_cell_formula_units_Z 1
loop_
_atom_type_symbol
_atom_type_radius_bond
Li 1.23
S 1.04
Ti 1.4
loop_
_atom_site_label
_atom_site_type_symbol
_atom_site_fract_x
_atom_site_fract_y
_atom_site_fract_z
Li1 Li 0.988050806591502 0.990131573271804 1.16958232444791E-02
Li2 Li 3.35019583167642E-03 0.99978534401799 0.3218378122706
Li3 Li 0.997400777136704 0.335248398459501 0.666803787942405
Li4 Li 0.993305262915361 0.6741506372854 8.87541728086313E-03
Li5 Li 0.997159697839832 0.668304367640801 0.330991425654606
Li6 Li 0.993853378859383 0.671195945339601 0.6684335114157
Li7 Li 0.333911954459701 6.95176179580452E-03 0.993239131075606
Li8 Li 0.3292838529831 1.09562736018344E-03 0.6713188531103
Li9 Li 0.3468234054033 0.339661281466 0.997453491900863
Li10 Li 0.3449787413269 0.337538330807401 0.3319623887435
Li11 Li 0.3315854015089 0.663890080349501 0.674318857118998
Li12 Li 0.672875453960901 1.56873294144245E-03 2.27090284620937E-03
Li13 Li 0.677806745036601 0.3503350409733 0.988485158896353
Li14 Li 0.6765861881544 0.326420829591201 0.3259691335101
Li15 Li 0.671341042218301 0.3357561891142 0.658198676789603
Li16 Li 0.6669837228548 0.6666503306486 1.35612990494068E-03
Li17 Li 0.677188902645901 0.665003745291601 0.329062592934597
Li18 Li 0.660821673631101 0.664104620928601 0.676382492907896
S1 S 0.1786207750744 0.1714760780881 0.1710103587513
S2 S 0.1580701176319 0.1478460775933 0.499383667828
S3 S 0.1745077918462 0.1957169368093 0.817690334650499
S4 S 0.1761284913705 0.4988833829368 0.1682892246132
S5 S 0.1816186879289 0.507033322262601 0.498394759267
S6 S 0.1706161986887 0.488090336278001 0.837493677739003

S7   S   0.1879663374606   0.8148139429446   0.1601933664675
S8   S   0.183830417353601   0.836521944022301   0.479403916813502
S9   S   0.1545152548922   0.8403373578599   0.831605740310696
S10   S   0.4929740502383   0.143441502483801   0.1952093739138
S11   S   0.517909232379401   0.154216867231101   0.493325208175799
S12   S   0.5068937008344   0.175468946039001   0.8020666109368
S13   S   0.4884607384237   0.5161580158548   0.1664220666335
S14   S   0.4850712299128   0.4958284512332   0.495479253237303
S15   S   0.488578705196401   0.5175485349273   0.847912829741703
S16   S   0.4875676962986   0.828555742461201   0.1812680573304
S17   S   0.493126607375001   0.8361714736661   0.496599386206999
S18   S   0.505373819414101   0.8450202289817   0.827836695920497
S19   S   0.8577099770596   0.180315958107801   0.1403042975304
S20   S   0.8528302128451   0.159926245610801   0.496401851664898
S21   S   0.836722352105501   0.164396227497801   0.827889873310103
S22   S   0.867043163910405   0.4774600322407   0.160961859746198
S23   S   0.849729706449401   0.482001715737201   0.487063284844804
S24   S   0.840779547888701   0.504448463307   0.834584265006497
S25   S   0.818883026888601   0.8412443744067   0.184540757957402
S26   S   0.813317107991201   0.854222026060801   0.496352996230101
S27   S   0.832619844618202   0.862725902792801   0.803775921820603
Ti1   Ti   0.98530889646541   9.34363650479652E-03   0.698601214760297
Ti2   Ti   0.980775973298685   0.3407139477434   0.983736509069928
Ti3   Ti   0.979183159655009   0.3180799190236   0.360106954066205
Ti4   Ti   0.335105661568201   0.995449928332508   0.312654303903101
Ti5   Ti   0.3202996000883   0.353355297389201   0.683029908264298
Ti6   Ti   0.324363797178701   0.629792337615701   3.04775500547505E-02
Ti7   Ti   0.322313575290801   0.6635940381668   0.3427904497497
Ti8   Ti   0.646262175210801   0.96536160101287   0.352341453414701
Ti9   Ti   0.641615165811201   0.98664636948794   0.676146432519498
#END

1435
_chemical_formula_sum 'Li18 S27 Ti9'
_chemical_formula_moiety
;
Li18 S27 Ti9
;
loop_
_publ_author_name
Ugliengo
_cell_volume 1030.1946
_symmetry_space_group_name_H-M 'P 1'
_symmetry_cell_setting triclinic
_symmetry_Int_Tables_number 1
loop_
_symmetry_equiv_pos_site_id
_symmetry_equiv_pos_as_xyz
1 x,y,z
_cell_length_a 10.9056424
_cell_length_b 12.2727955
_cell_length_c 11.1307226
_cell_angle_alpha 56.953357
_cell_angle_beta 63.427827
_cell_angle_gamma 57.758904
_cell_formula_units_Z 1
loop_
_atom_type_symbol
_atom_type_radius_bond
Li 1.23

S 1.04
Ti 1.4
loop_
_atom_site_label
_atom_site_type_symbol
_atom_site_fract_x
_atom_site_fract_y
_atom_site_fract_z
Li1   Li   7.22445954412059E-03   0.3241112163366   8.95151975746269E-03
Li2   Li   3.70523268756502E-02   0.3067538218045   0.299126806579296
Li3   Li   9.93300864344167E-03   0.5877132401114   1.3309413280968E-03
Li4   Li   9.34729288146415E-03   0.5979989927129   0.312221748546796
Li5   Li   3.33161809340511E-03   0.7377274131482   0.658603082050197
Li6   Li   0.3474327907523   1.72988322637206E-03   0.998237411204421
Li7   Li   0.358115981156001   0.94867005832958   0.661683464473397
Li8   Li   0.3363840838409   0.345914099406   4.36487830278372E-03
Li9   Li   0.291811421477701   0.3190534598189   0.359139511845795
Li10   Li   0.337318817630401   0.3370968391385   0.664733739170897
Li11   Li   0.306787657425401   0.6046769517668   0.002304004515592
Li12   Li   0.3290699456361   0.5907518705569   0.689518277359594
Li13   Li   0.676611957238102   0.97923833737032   0.331015542698295
Li14   Li   0.6762526302921   2.26927192906203E-02   0.638787131329095
Li15   Li   0.6441984555187   0.3626410433698   2.57583345796249E-03
Li16   Li   0.678513676882401   0.3910034715013   0.682825030183199
Li17   Li   0.666804888244501   0.6614604073358   0.369358469783898
Li18   Li   0.630917691250701   0.6384974408307   0.699440565040198
S1   S   0.173918227712501   0.127356540309   0.181847353253996
S2   S   0.180309523473101   0.1186450260106   0.501392916100396
S3   S   0.1209822700689   0.2292068918184   0.789959328616793
S4   S   0.162475166681701   0.4692010505059   0.168193184523595
S5   S   0.174886061287601   0.5445088566444   0.457022939589396
S6   S   0.130560796459101   0.5015255568931   0.798282440654793
S7   S   0.1719207133247   0.8693038062227   0.156643449945496
S8   S   0.212492718920001   0.8226848037911   0.462613767567395
S9   S   0.152128050601801   0.875342274194599   0.853301572946694
S10   S   0.5033703708274   0.1165889446547   0.160051159497795
S11   S   0.5181116732011   0.2270778094474   0.442247565054096
S12   S   0.5068300654994   0.1429618513084   0.831620140256697
S13   S   0.474040212501401   0.5492136545302   0.171239460071296
S14   S   0.5114847122329   0.502194459457099   0.487617998180494
S15   S   0.483824063533601   0.492112688182101   0.843188335799792
S16   S   0.562785729866401   0.8111776964372   0.137875615783396
S17   S   0.510028188080701   0.8303977607073   0.488718705345698
S18   S   0.5191439697364   0.846396458472101   0.828293803369896
S19   S   0.8523177864909   0.1478754608137   0.139169402067595
S20   S   0.8596201788144   0.1576959528995   0.473904137376793
S21   S   0.817847437578401   0.1432516453084   0.847258596281094
S22   S   0.814410808974501   0.4764823496129   0.176091551782896
S23   S   0.8583044660267   0.5022165702321   0.547763989863293
S24   S   0.786605714286901   0.515668268473399   0.876017180536592
S25   S   0.852741331799405   0.8416584516951   0.165561113140191
S26   S   0.856404257840006   0.8707052016617   0.48485992169509
S27   S   0.865925946091397   0.860110694549899   0.824774391035601
Ti1   Ti   0.979512518184268   0.97795025883963   3.39345232446598E-02
Ti2   Ti   0.98945472402905   4.43532302167115E-03   0.326921819369295
Ti3   Ti   0.979449435015961   1.50069114889602E-02   0.709765276849594
Ti4   Ti   0.997299838996424   0.456514275518699   0.695600750368895
Ti5   Ti   0.333605730625901   0.999588567358608   0.351862451774199
Ti6   Ti   0.3124054654412   0.6677724209504   0.341907506487695
Ti7   Ti   0.646005755504101   2.08115262462021E-03   0.993314568205718

Ti8   Ti   0.6470305059801   0.3598298954504   0.358938058585496
Ti9   Ti   0.634656880926701   0.6675292038538   8.0270671534393E-03
#END

218
_chemical_formula_sum 'Li18 S27 Ti9'
_chemical_formula_moiety
;
Li18 S27 Ti9
;
loop_
_cell_volume 1002.549
_symmetry_space_group_name_H-M 'P 1'
_symmetry_cell_setting triclinic
_symmetry_Int_Tables_number 1
loop_
_symmetry_equiv_pos_site_id
_symmetry_equiv_pos_as_xyz
1 x,y,z
_cell_length_a 11.2292252
_cell_length_b 11.1455588
_cell_length_c 11.876267
_cell_angle_alpha 50.429829
_cell_angle_beta 71.109759
_cell_angle_gamma 61.072522
_cell_formula_units_Z 1
loop_
_atom_type_symbol
_atom_type_radius_bond
Li 1.23
S 1.04
Ti 1.4
loop_
_atom_site_label
_atom_site_type_symbol
_atom_site_fract_x
_atom_site_fract_y
_atom_site_fract_z
Li1   Li   0.05497641592489   0.94706313591269   0.332357121222504
Li2   Li   0.8709980191902   0.1182735891469   0.677510952155604
Li3   Li   5.75916746115901E-02   0.2519577410857   0.337433205402204
Li4   Li   0.8494179305957   0.4904813093676   0.664582833649804
Li5   Li   0.9686543493533   0.6997358469956   0.998529974450036
Li6   Li   0.8701750837998   0.7997174779041   0.650864884834004
Li7   Li   0.2947644521949   2.52752684453097E-02   0.985857213518834
Li8   Li   0.3838373539679   1.47017011703497E-02   0.245167832513604
Li9   Li   0.2554489436923   6.29444317390497E-02   0.680287522282004
Li10   Li   0.2726238969499   0.4005006732674   0.973148424717844
Li11   Li   0.4203433411862   0.2561122048637   0.310656170658004
Li12   Li   0.3139464805477   0.6981389112011   2.13029419433437E-02
Li13   Li   0.4187619143864   0.565578655674   0.339722459124704
Li14   Li   0.5350715055901   8.08682978569397E-02   0.723995562083404
Li15   Li   0.6389836703477   0.3641418119305   2.24586506168037E-02
Li16   Li   0.5703621717797   0.3942130047371   0.720051241851204
Li17   Li   0.6132656084364   0.6973262720471   2.12279108243437E-02
Li18   Li   0.555030204169   0.7613134674241   0.749897384126604
S1   S   0.1553022531295   0.184475329466   0.138481332033604
S2   S   0.3026763083251   2.71869663253897E-02   0.479810501311104
S3   S   7.13697734874901E-02   0.2526515587912   0.833301799360204
S4   S   0.1765710434716   0.4863187220327   0.183288728663904

S5 S 0.2582497511382 0.3883046795668 0.506248150266504
S6 S 7.99692258393901E-02 0.5754942909298 0.807854962227204
S7 S 0.1511933534453 0.8241243258566 0.165427907030204
S8 S 0.2878440703406 0.6938218756811 0.493588414514404
S9 S 8.04934210103201E-02 0.9219554758344 0.838690547823603
S10 S 0.5556673411384 0.1263136110863 0.157781944346304
S11 S 0.6362988937408 3.07304046885096E-02 0.486149428230504
S12 S 0.3981368818432 0.2676864047999 0.815742145705204
S13 S 0.5334242550773 0.4497185647895 0.199672772597604
S14 S 0.6345970247807 0.372319097321 0.495464109493504
S15 S 0.4271762797035 0.5841386384498 0.815240546980004
S16 S 0.5463135770114 0.8119397498244 0.177348754794604
S17 S 0.6298132290007 0.7052672375896 0.490867417877504
S18 S 0.4197298419081 0.90169668768409 0.854659504960304
S19 S 0.8437253118376 0.1507264194846 0.180104272890104
S20 S 0.92375287759757 5.95967596723197E-02 0.492394171200204
S21 S 0.768542599789 0.2148458150472 0.863660007640404
S22 S 0.8361658511355 0.4813992971479 0.184919479916304
S23 S 0.93690720100798 0.4143755678426 0.473088822408804
S24 S 0.7745273703993 0.5575359085463 0.857670613825504
S25 S 0.8646635912806 0.8180821989712 0.168022975565004
S26 S 0.94079196915638 0.7214989421422 0.468831907963304
S27 S 0.7424575935546 0.92323197117068 0.861331779060303
Ti1 Ti 0.990528698248415 1.80956909332497E-02 0.970884273427994
Ti2 Ti 0.98512079004208 0.3688356646134 0.963420274980104
Ti3 Ti 8.72064889095601E-02 0.571569412689 0.332625631028204
Ti4 Ti 0.2232711232336 0.462868314453 0.669878484317704
Ti5 Ti 0.399858476361 0.7566282487063 0.576167579260204
Ti6 Ti 0.6798168496184 0.98602046611566 2.48771969754737E-02
Ti7 Ti 0.7339775345323 0.94128466383918 0.342098488938304
Ti8 Ti 0.750076917578 0.2586107343178 0.325510342719104
Ti9 Ti 0.7295292146043 0.5623065028212 0.349842443692404
#END

1097
_chemical_formula_sum 'Li18 S27 Ti9'
_chemical_formula_moiety
;
Li18 S27 Ti9
;
loop_
_cell_volume 1046.5927
_symmetry_space_group_name_H-M 'P 1'
_symmetry_cell_setting triclinic
_symmetry_Int_Tables_number 1
loop_
_symmetry_equiv_pos_site_id
_symmetry_equiv_pos_as_xyz
1 x,y,z
_cell_length_a 12.2638561
_cell_length_b 11.1995018
_cell_length_c 10.9625801
_cell_angle_alpha 66.165976
_cell_angle_beta 57.577622
_cell_angle_gamma 56.435745
_cell_formula_units_Z 1
loop_
_atom_type_symbol
_atom_type_radius_bond
Li 1.23

S 1.04
Ti 1.4
loop_
_atom_site_label
_atom_site_type_symbol
_atom_site_fract_x
_atom_site_fract_y
_atom_site_fract_z
Li1 Li 0.988300136500462 0.987762124430601 7.8973145680484E-03
Li2 Li 3.38295576053791E-02 0.994017740141768 0.337206949308503
Li3 Li 3.46919806417199E-02 7.90016162521825E-03 0.658028338527203
Li4 Li 2.07004718400301E-02 0.333047266042601 3.31836774487227E-02
Li5 Li 0.967162530295261 0.2988924468236 0.750789824897908
Li6 Li 4.39111239185698E-02 0.717616865514701 0.925581392025562
Li7 Li 0.0103192501887 0.742441043196901 0.245715009996303
Li8 Li 2.70487531742393E-02 0.722505125463701 0.5984539703095
Li9 Li 0.291977874252698 0.993858588596902 1.29851865323637E-02
Li10 Li 0.308061540909798 0.991526584583481 0.321748405304002
Li11 Li 0.335750470811899 0.7100319545808 0.96464361218454
Li12 Li 0.298970697956598 0.718076506970301 0.290913756978604
Li13 Li 0.349868323937198 0.7116725737878 0.579484467442209
Li14 Li 0.734105551982597 6.95389218847791E-04 0.990580821531324
Li15 Li 0.6837392119695 0.967765896629091 0.315115522964201
Li16 Li 0.645680916145697 0.310917035308201 1.11373418177743E-02
Li17 Li 0.702276231073097 0.2993713193021 0.694159060523904
Li18 Li 0.747336610818 0.658842526773601 0.289625677465901
S1 S 0.1219628868829 0.200981793096401 0.217189797002903
S2 S 0.196770185155299 0.1647947997868 0.503090443340904
S3 S 0.183577299138999 0.1732871082998 0.822073804635399
S4 S 0.179582802441099 0.473520294734 0.224846228602304
S5 S 0.173458472149099 0.4674808040731 0.554576509525003
S6 S 0.170955082800799 0.4690878352309 0.893014459358402
S7 S 0.159482044282899 0.8519800923339 0.148792210113103
S8 S 0.1676849205661 0.848238552377301 0.4899421474045
S9 S 0.208094436628399 0.8519644655099 0.792791204983305
S10 S 0.478347162556198 0.1655000474049 0.164380977790605
S11 S 0.490469476932798 0.1690682549496 0.493257781182004
S12 S 0.459829943425498 0.1601204534822 0.845617516588208
S13 S 0.473744873875898 0.463078352805101 0.222269300846604
S14 S 0.474300275344498 0.455168796366801 0.551406609977106
S15 S 0.468439997652997 0.468060549595401 0.882102395341106
S16 S 0.484326351017597 0.822498143520801 0.125000229465305
S17 S 0.532437815922498 0.815086791270101 0.494201746856508
S18 S 0.495503137286898 0.8699705140361 0.783238010140604
S19 S 0.806686571284397 0.160617861593101 0.184870007171605
S20 S 0.771600861458997 0.139956462899801 0.522838556846906
S21 S 0.844838402674596 0.1577708173993 0.832404285723705
S22 S 0.769430619824495 0.4898884896069 0.176200781135806
S23 S 0.826645451702193 0.4754419294864 0.502100536828206
S24 S 0.743876851880297 0.549623320800701 0.833400144118609
S25 S 0.8608217477047 0.813560265817 0.134166119223304
S26 S 0.822477544006797 0.815591439573601 0.503358427475108
S27 S 0.803563077188801 0.837068177107501 0.823702308687304
Ti1 Ti 0.0635876222812 0.328258220165401 0.371652416429703
Ti2 Ti 0.322184980120899 0.990254360306397 0.679493055489702
Ti3 Ti 0.356774101570099 0.3308311487326 1.12081443911637E-02
Ti4 Ti 0.345973790418998 0.3306556546595 0.361467908835204
Ti5 Ti 0.320666029152999 0.3502618708043 0.690164339167702
Ti6 Ti 0.6708965651392 0.98941885830111 0.683544913551004
Ti7 Ti 0.646939306823897 0.316145094008701 0.333776238340004

Ti8	Ti	0.695323327889898	0.671414067109301	0.999825869923822
Ti9	Ti	0.685014750801798	0.696413163761701	0.620784243677308

#END
